# ActVI-ORFA directs metabolic flux towards actinorhodin by preventing intermediate degradation

**DOI:** 10.1371/journal.pone.0308684

**Published:** 2024-08-09

**Authors:** Xuechen Zhu, Rongbin Wang, Vilja Siitonen, Nemanja Vuksanovic, Nicholas R. Silvaggi, Charles E. Melançon III, Mikko Metsä-Ketelä

**Affiliations:** 1 Department of Chemistry and Chemical Biology, University of New Mexico, Albuquerque, New Mexico, United States of America; 2 Department of Life Technologies, University of Turku, Turku, Finland; 3 Department of Chemistry and Biochemistry, University of Wisconsin-Milwaukee, Milwaukee, Wisconsin, United States of America; Agricultural University of Athens: Geoponiko Panepistemio Athenon, GREECE

## Abstract

The biosynthetic pathway of actinorhodin in *Streptomyces coelicolor* A3(2) has been studied for decades as a model system of type II polyketide biosynthesis. The actinorhodin biosynthetic gene cluster includes a gene, *actVI-orfA*, that encodes a protein that belongs to the nuclear transport factor-2-like (NTF-2-like) superfamily. The function of this ActVI-ORFA protein has been a long-standing question in this field. Several hypothetical functions, including pyran ring cyclase, enzyme complex stability enhancer, and gene transcription regulator, have been proposed for ActVI-ORFA in previous studies. However, although the recent structural analysis of ActVI-ORFA revealed a solvent-accessible cavity, the protein displayed structural differences to the well-characterized cyclase SnoaL and did not possess a DNA-binding domain. The obtained crystal structure facilitates an inspection of the previous hypotheses regarding the function of ActVI-ORFA. In the present study, we investigated the effects of a series of *actVI-orfA* test plasmids with different mutations in an established vector/host system. Time-course analysis of dynamic metabolism profiles demonstrated that ActVI-ORFA prevented formation of shunt metabolites and may have a metabolic flux directing function, which shepherds the flux of unstable intermediates towards actinorhodin. The expression studies resulted in the isolation and structure elucidation of two new shunt metabolites from the actinorhodin pathway. Next, we utilized computational modeling to probe the active site of ActVI-ORFA and confirmed the importance of residues R76 and H78 in the flux directing functionality by expression studies. This is the first time such a function has been observed for a member of NTF-2-like superfamily in *Streptomyces* secondary metabolism.

## Introduction

Microbial natural products have been instrumental in drug development and have led to the introduction of numerous antibiotics and anticancer agents into the clinic. Polyketides comprise chemically diverse structures with potent antimicrobial and anticancer activities [1, 2], which have yielded numerous widely used drugs and are an attractive family of lead compounds for drug development. Among the family of polyketides, type II polyketides (PK-IIs) are an important subclass of polyketides, which has contributed the antibiotic tetracycline [3] and anticancer agent doxorubicin [4] to the pharmaceutical industry.

Recent advancements in synthetic biology have allowed the engineering of biosynthetic pathways to further expand the chemical space of bioactive natural products [5, 6]. However, much of the progress in this area has been dependent on detailed understanding of the metabolic pathways responsible for the biosynthesis of the molecules of interest. This fundamental knowledge has allowed the development of bioinformatic tools for the rapid identification of unknown metabolic pathways from genome sequencing data and has made rational metabolic engineering approaches feasible [7, 8].

PK-IIs are typically polyphenolic and polycyclic compounds that harbor at least one aromatic ring and the shared biosynthetic logic of these molecules has been elucidated in detail [9, 10]. Briefly, the biosynthesis involves the generation of a poly-β-ketone chain by a heterodimeric ketosynthase (KSα/β) and an acyl carrier protein (ACP). The polycyclic aglycone units are formed by an optional 9-ketoreductase (C9KR) with a combination of aromatase/cyclase (AroCyc) and cyclase (Cyc) enzymes for the regiospecific cyclization and aromatization. The aromatic polycyclic intermediate resulting from these enzymatic reactions is commonly referred to as a core structure, which is further modified by a diverse array of tailoring enzymes, including oxidases, reductases, and group transfer enzymes, to produce the final product.

**Fig 1:**
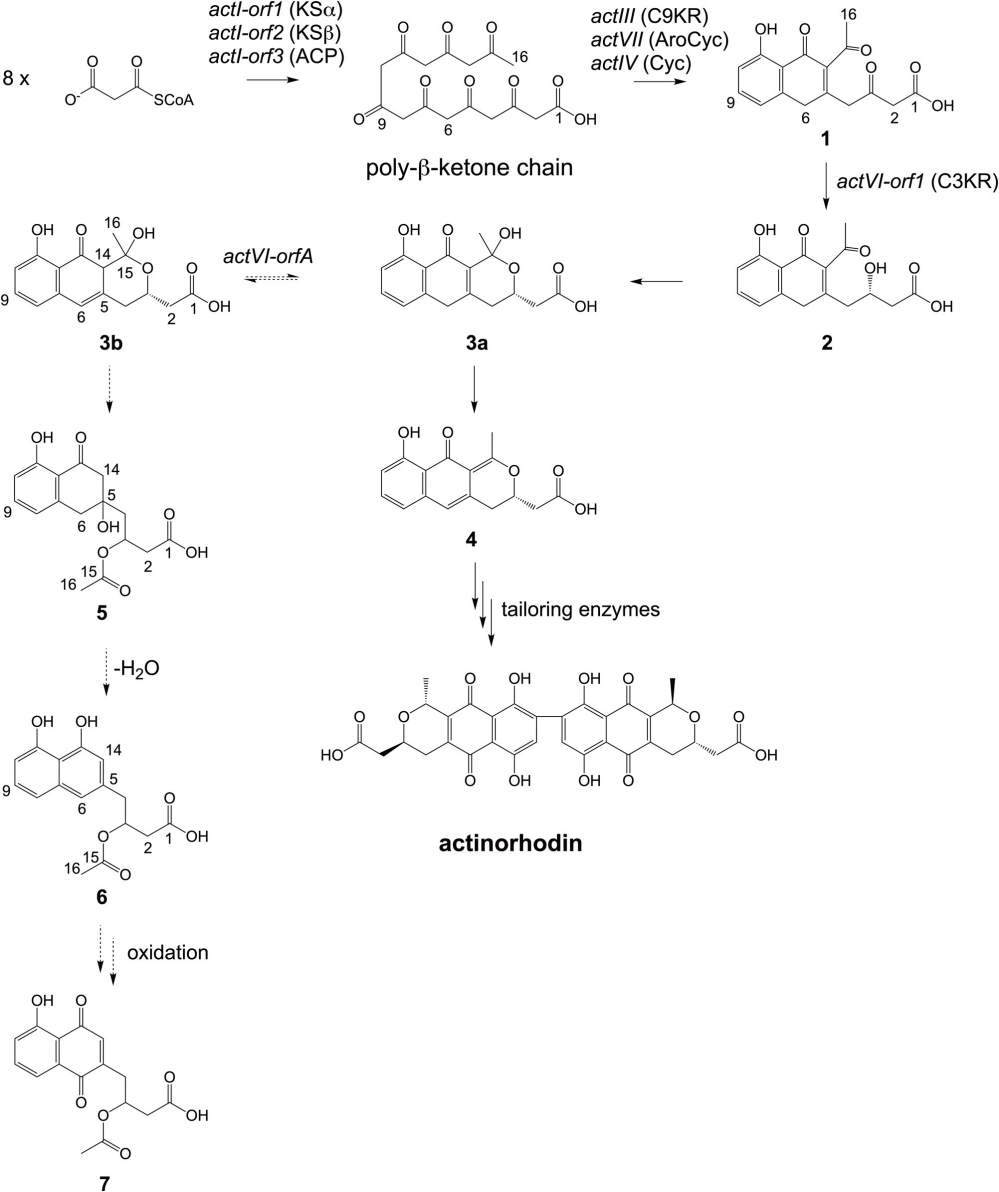
The biosynthesis of the actinorhodin core structure (*S*)-DNPA (4) in *S*. *coelicolor* A3(2). Dashed arrows show the shunt metabolism pathway to (*S*)-NHAB (**7**) [14, 15] through **5**/**6** elucidated in the present study. The biosynthesis genes, the encoded proteins, and the important intermediates [11, 16–18] are annotated. Specifically, gene *actVI-orfA*, encoding the protein ActVI-ORFA, is annotated as directing the metabolic flux towards actinorhodin by interacting with **3b** based on this work.

Actinorhodin (Fig 1) is an archetypical PK-II compound produced by *Streptomyces coelicolor* A3(2) [11] that has served as a model system for biosynthetic studies of bacterial PK-IIs [12]. The core structure of actinorhodin, (*S*)-DNPA (4-dihydro-9-hydroxy-1-methyl-10-oxo-3-H-naphtho-[2,3-c]-pyran-3-(*S*)-acetic acid, **4** in Fig 1), was isolated from the culture medium of an engineered *S*. *coelicolor* strain that does not harbor genes encoding tailoring enzymes [13].

Investigations into the early steps of PK-II biosynthesis have been challenging due to the instability of pathway intermediates, but the steps in the formation of **4** in actinorhodin biosynthesis have been elucidated in detail (Fig 1). One remaining question has been the role of ActVI-ORFA, which is a member of nuclear transport factor-2-like (NTF-2-like) superfamily. In an early study [19], ActVI-ORFA, along with ActVI-ORF3, was proposed to be involved in the pyran ring closure in the formation of **4** (the step from **2** to **3a** in Fig 1). However, subsequent studies suggested that pyran ring closure and dehydration might occur spontaneously [13]. The observation that ActVI-ORFA homologs are also present in biosynthetic pathways of pyran ring-free PK-II compounds [20–22] led to a proposal that ActVI-ORFA may have a more general, non-enzymatic, role on the pathway. Inactivation of *actVI-orfA* in the wild type *S*. *coelicolor* led to identification of a shunt metabolite (*S*)-NHAB (1,4-naphthoquinone-8-hydroxy-3-[3(*S*)-acetoxy-butyric acid], **7**) [14, 15]. The compound was presumed to result from the isomerization of **3a** to **3b**, followed by the cleavage of the carbon-carbon bond between C14 and C15 (labeled with dashed arrows in Fig 1), which was regarded as a retro-Claisen type reaction and may occur when the dehydration of the pyran ring is interrupted [16, 23]. Therefore, ActVI-ORFA was suggested to be an accessory protein, which enhances the stability of a putative multi-enzyme complex where closely related tailoring enzymes co-operate for efficient production of actinorhodin [16]. In a further functional analysis [24], this protein was proposed to be a transcriptional regulatory factor promoting the expression of a key actinorhodin ketoreductase gene. This idea was adopted in recent experiments with ActVI-ORFA and its homolog Med-ORF10 from the medermycin pathway [25, 26], but the putative target promoter could not be identified in either study.

The structure of ActVI-ORFA was recently determined using X-ray crystallography [27], which revealed a fold typical for NTF-2-like proteins that consists of three helices and a partial β-barrel enclosing a solvent-accessible cavity. However, structural comparison against a well-characterized PK-II cyclase, SnoaL involved in anthracycline biosynthesis [28], revealed a highly dissimilar active site architecture. Also, unlike another characterized polyketide cyclase AknH [29], residues in the solvent-accessible cavity in ActVI-ORFA do not align well with those in the active site of SnoaL, including the conserved important residues H107, H119, and D121. Therefore, the protein is very unlikely to function as a cyclase. Similarly, the active site cavity of ActVI-ORFA bears little similarity to the cofactor-independent monooxygenases SnoaL2 and AclR [30, 31], nor does the protein contain a nucleic acid-binding domain commonly required for transcriptional regulators [27].

The paradox between the proposed function and structure of ActVI-ORFA prompted us to re-investigate the role of ActVI-ORFA in actinorhodin biosynthesis. Here we leveraged gene cassettes harboring early actinorhodin biosynthetic genes that were constructed in a “bottom-up” manner in a previously established host/vector system to minimize metabolism background [32]. Analysis of metabolism profiles of *actVI-orfA* mutant strains led to the identification of two new actinorhodin shunt metabolites. Dynamic metabolism time-course analysis indicated that the formation of shunt metabolites increased in the absence of *actVI-orfA*. Molecular modeling and docking analysis were used to identify important functional residues of ActVI-ORFA. The obtained results suggest that ActVI-ORFA may function to direct the metabolic flux towards actinorhodin in its biosynthesis.

## Materials and methods

### Construction of plasmids

The *actVI-orfA* gene variants carrying the site-directed mutations were synthesized at Genewiz (Suzhou, China). The sequences of the synthesized DNA are shown in S1 Appendix. Primers were synthesized at Integrated DNA Technologies (Coralville, IA USA). PCR reactions were carried out with the Phusion High-Fidelity PCR Kit from Thermo Fisher Scientific Inc. (Waltham, MA USA). The concentrations of template, primers, polymerase, dNTPs, and buffer recommended in the manufacturer’s manual were used in PCR reactions. Information about other reagents, kits, and instruments is provided in S1 Appendix.

For constructing plasmid pXZ14, the sequences of *actI-orf1*, *actI-orf2*, *actI-orf3*, *actIV*, and *actVII* were amplified from *S*. *coelicolor* A3(2) genome with primers act_KSa_up (forward) and act_KSa_dn (reverse), primers act_KSb_up (forward) and act_KSb_dn (reverse), primers act_ACP_up (forward) and act_ACP_dn_2 (reverse), primers act_Aro_up (forward) and act_Aro_dn (reverse), and primers act_Cyc5_up (forward) and act_Cyc5_dn_2 (reverse), respectively. The sequence of *actVI-orf1* was amplified from pXZ11 [32] with primers act_C3KR_up_2 (forward) and act_Cyc3_dn (reverse) and primers act_C3KR_up (forward) and act_C3KR_dn_2 (reverse).

The amplicons were assembled using COE-PCR described before [32]. Briefly, the sequences of the *E*. *coli* replication origin colE1 and the selective marker ampicillin resistance gene were amplified from plasmid COE-PCR_act_minimal_PKS (generated by Zhu in Melançon Lab) with primers act_colE1_up_2 and act_ampR_dn_2. Then an overlapping extension PCR was performed with all amplicons to generate a plasmid carrying the assembled gene cassette. The product plasmid was amplified in *E*. *coli* host. The gene cassette was then cut from the plasmid with EcoRI and XbaI.

Additionally, the sequence of *actIII* was cut from plasmid COE-PCR_act_Part_1 (generated by Zhu in Melançon Lab) with BsrGI and EcoRI. The vector pXZ4 [32] was digested with BsrGI and XbaI. A three-way ligation was carried out to ligate these three fragments to generate the plasmid pXZ14.

For constructing the pRW plasmids carrying *actVI-orfA* variants, the synthesized DNA was subcloned into Genewiz cloning plasmid pUC-GW-Kan with EcoRV for amplification. The amplified *actVI-orfA* gene variants were cloned into pXZ14 with XbaI and HindIII.

The information about the bacterial strains and culture conditions used in cloning are provided in S1 Appendix. The sequences of primers and the maps of plasmids are shown in S1 Appendix.

### Metabolism analysis

Three hundred μL of the 20% glycerol stock of each transformant strain was transferred to 15 mL of R5 medium in a 125 mL Erlenmeyer flask and was grown at 30°C, 250 rpm for five days. Cells were harvested with centrifugation (4000 *g*, 10 min, room temperature), washed twice with 15 mL of fresh R4 medium, resuspended in 10 mL of fresh R4 medium, and manually homogenized with a sterilized glass tissue grinder (WHEATON, DWK Life Sciences, USA). After the homogenization, the cell density of the culture was determined based on OD600. Then the equal amounts of cells from each culture were transferred to 100 mL of R4 medium in 500 mL Erlenmeyer flasks. Three replicates were made. The R4 media were then grown at 28°C, 180 rpm for eight days.

Two mL of the medium was separated from each culture every two days (48 hours). Specifically, the day 0 samples were prepared by separating the medium before cultivation. The separated medium was sampled immediately. It was first clarified by centrifugation (14,000 *g*, 10 min, room temperature), then acidified to pH 3.0 using 2 N HCl and extracted with 1 volume of ethyl acetate three times. The extract was dried with rotovap and overnight vacuum and was stored at -20°C until being analyzed.

For metabolism analysis, the dried extract was dissolved with 200 μL of 50% acetonitrile and the resultant sample solution was clarified by centrifugation (14,000 *g*, 10 min, room temperature). Ten μL of the clarified sample solution was injected into a C18 column and analyzed with high-performance liquid chromatography (HPLC) using the following program: 5% solvent B for 1 minute, 5% to 98% solvent B over 18 minutes, 98% solvent B for 3 minutes, where solvent A was ultrapure water with 0.1% formic acid and solvent B was acetonitrile with 0.1% formic acid. The flow rate was 0.6 mL/min. The detector wavelength was 254 nm. Specifically, the analysis of M1152::pXZ11 and M1152::pXZ14 was performed with a Kromasil C18 column (Kromasil octadecyl phase, 5 μm, 150 × 3 mm) on a Dionex Ultimate 3000 HPLC system and the analysis of pRW transformant strains was performed with a Kinetex Core-Shell C18 column (Phenomenex, 2.6 μm, 100 × 4.6 mm) on a Shimadzu SCL-10Avp HPLC system.

### Production and isolation of metabolites

For characterization, compounds **5** and **6** were isolated from a large-scale culture of M1152::pXZ14. To grow the bacterial culture, 300 μL of the glycerol stock was enriched in 15 mL of R5 medium in a 125 mL Erlenmeyer flask at 30°C, 250 rpm for five days. Five mL of the resultant culture was transferred to 200 mL of R5 medium in a 1 L Erlenmeyer flask for another five-day propagation. Forty mL of the propagated culture was used to inoculate each of five 400 mL of R4 medium in 2 L Erlenmeyer flasks, which were then grown at 28°C, 180 rpm for seven days. After the cultivation, cells were pelleted by centrifugation (4000 *g*, 10 min, room temperature). The supernatant medium was collected and further clarified with vacuum filtration. The clarified medium was acidified to pH 3.0 using 2 N HCl and extracted three times with 1 volume of ethyl acetate. The extract was dried and re-dissolved in 10 mL of 50% acetonitrile. The solution was clarified by centrifugation (14,000 *g*, 10 min, room temperature) and filtration using a 0.20 μM PTFE filter.

To isolate the compounds, the clarified solution was injected into a Kromasil C18 column (Kromasil octadecyl phase, 5 μm, 250 × 10 mm) on a Dionex Ultimate 3000 HPLC instrument. The crude isolate obtained in the first-round separation was dried and re-dissolved in DMSO and was refined in the second round. Solvents A and B as same as those used in the metabolism analysis were used in this two-round separation. The flow rate was 3.5 mL/min, and the detector wavelength was 254 nm.

The HPLC program used in the first round was: 40% Solvent B for 4 min, 40% to 98% Solvent B over 12 min, and 98% Solvent B for 3 min. The retention time (minutes) of **5**/**6** under this condition was 6.797.

The HPLC program used in the second round was: 5% Solvent B for 1 min, 5% to 98% Solvent B over 18 min, and 98% Solvent B for 3 min. The retention time (minutes) of **5**/**6** under this condition was 13.133.

The isolated compound was dried and stored at -20°C until being analyzed. Its weight was determined by the weight difference between the empty and the loaded container vial. In the present study, compounds **5**/**6** were isolated from the culture medium of M1152::pXZ14 at a yield of ~3.2 mg/L, and ~9.3 mg of the compound mixture was isolated for characterization.

### Characterization of compounds

Compound samples were dissolved with methanol for high-resolution mass spectrometry (HRMS) analysis. The mass spectra data was collected in the negative ionization mode (ESI).

The HRMS analysis of **5**/**6** was performed at a Waters/Micromass LCT Premier mass spectrometer facility at University of New Mexico.

The HRMS analysis of compound X was performed with a Waters ACQUITY Rda Detector with a Xbrige BEH C18 column (130 Å, 5 μm, 4.6 × 30 mm) at University of Turku. The mobile phase A was ultrapure water, 0.1% formic acid; the mobile phase B was methanol, 0.1% formic acid.

For nuclear magnetic resonance (NMR) analysis, the compound sample was dissolved with DMSO-d6 (Eurisotop). The NMR analysis was performed with the Bruker AVANCE-III NMR systems at University of Turku. 1D NMR data was collected using a 500 MHz NMR with a liquid nitrogen-cooled Prodigy BBO (CryoProbe) (^1^H and ^13^C) and a 600 MHz NMR with a liquid nitrogen-cooled Prodigy TCI (inverted CryoProbe) (^1^H). 2D NMR data (COSY, HSQC, and HMBC) was collected using a 600 MHz NMR with a liquid nitrogen-cooled Prodigy TCI (inverted CryoProbe).

### Molecular docking analysis of ActVI-ORFA

The PDB file of ActVI-ORFA (PDB ID: 5bka) was downloaded from Protein Data Bank (rcsb.org). The PDB file contains six chains (A–F). Chain E was separated and used in the docking studies. The PDB file of the ligand **3b** was generated using OpenBabel (http://www.cheminfo.org/Chemistry/Cheminformatics/FormatConverter/index.html#) with the 3D structural features added. The files for docking were prepared with the software MGLTools Version 1.5.6 (https://ccsb.scripps.edu/mgltools/downloads/). The docking was carried out with the AutoDock Suite v4.2.6 (https://autodock.scripps.edu/download-autodock4/). A docking grid covering the complete protein molecule was computed with AutoGrid. The docking calculation was carried out with AutoDock. The docking model was viewed and analyzed with PyMol v2.5.3 (https://pymol.org/2/).

## Results

### Expression studies of *actVI-orfA* in *S*. *coelicolor* M1152

**Fig 2:**
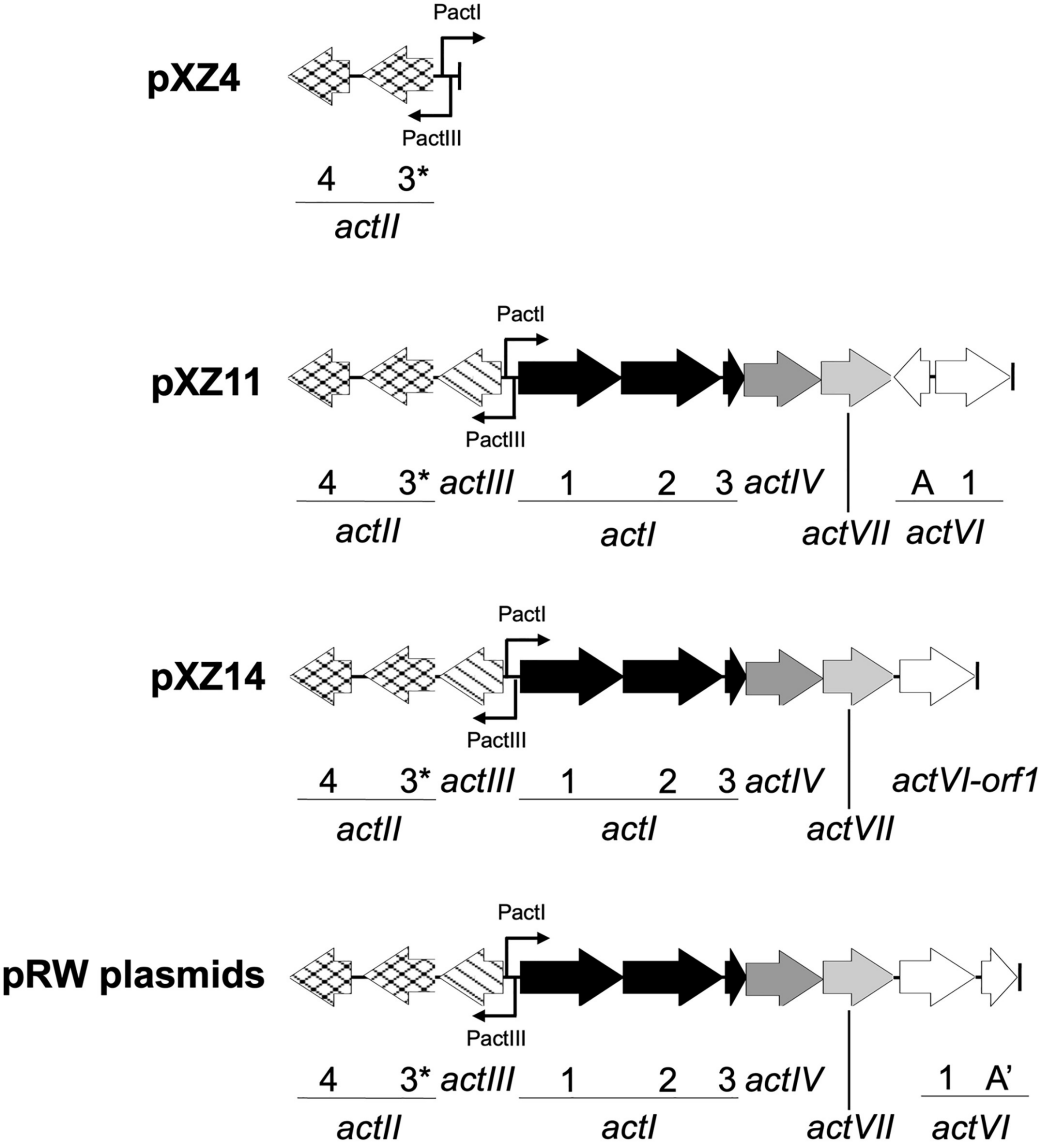
The gene cassettes in plasmids. The truncated *actII-orf3* guaranteeing the completion of the PactI system [32] is labeled with the asterisk, and A’ indicates the *actVI-orfA* gene variants.

**Fig 3:**
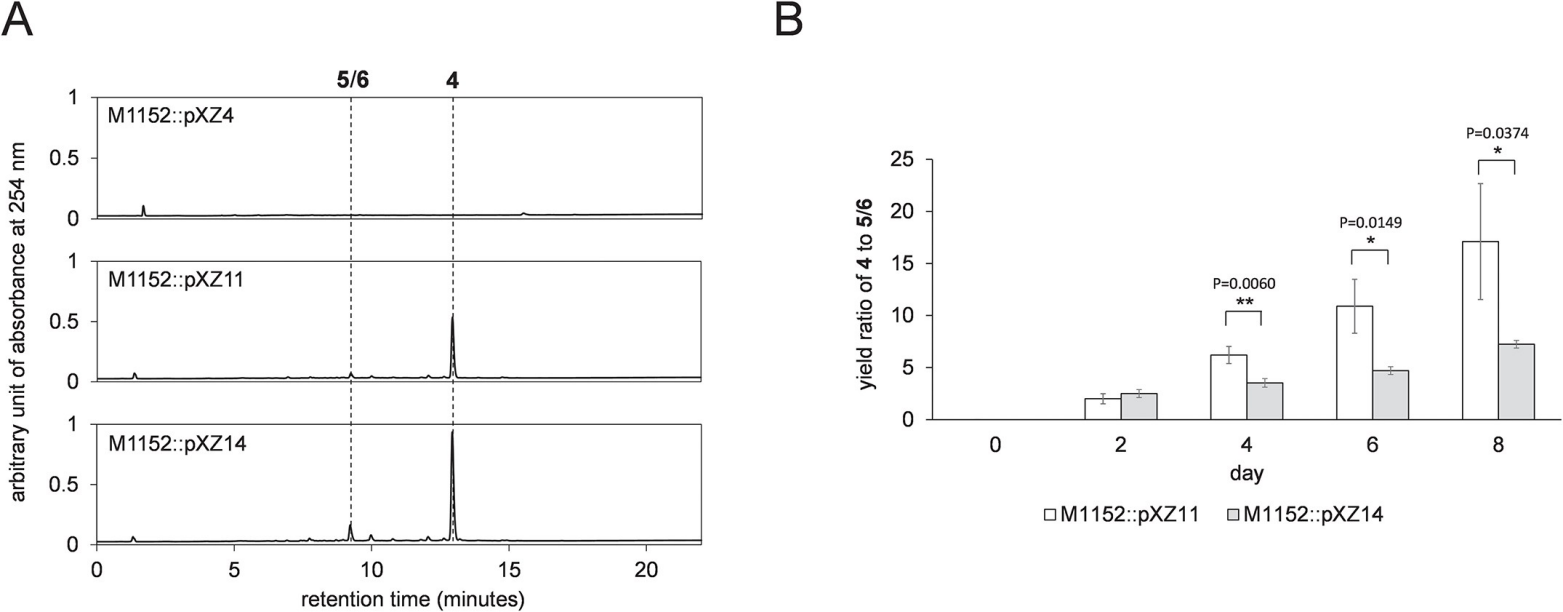
The metabolism analysis of *actVI-orfA* mutants. (A) The metabolic profiles of transformant strains M1152::pXZ4, M1152::pXZ11, and M1152::pXZ14 on day 8. Peaks of **4** and **5**/**6** are labeled. The retention times (minutes) of the metabolites are: **5**/**6** = 9.220 and **4** = 12.943. (B) The yield ratios of **4** to **5**/**6** in M1152::pXZ11 and M1152::pXZ14. Peak areas at 254 nm in HPLC spectra are used to represent the yields. Error bars represent the standard deviations (n = 3). The significant differences are evaluated with unpaired student t-test and labeled with * (P ≤ 0.05), ** (P ≤ 0.01), *** (P ≤ 0.001), and **** (P ≤ 0.0001) along with the calculated P values.

In a previous study [32], we assembled the actinorhodin biosynthesis genes *actI-orf1*, *actI-orf2*, *actI-orf3*, *actIII*, *actIV*, *actVII*, *actVI-orfA* and *actVI-orf1* in plasmid pXZ11 for production of (*S*)-DNPA (Fig 2). The genes were cloned into the vector plasmid pXZ4 (Fig 2), which served as a negative control. The functionality of these two plasmids has been validated in the host *S*. *coelicolor* M1152 [32]. Due to the adoption of a “bottom-up” method of plasmid construction and the use of an engineered host that has no active endogenous secondary metabolite biosynthesis pathways [33], the transformant strains M1152::pXZ4 and M1152::pXZ11 possessed clean metabolism profiles (see the HPLC traces in Fig 3A) and thus provided a useful system to investigate the function of ActVI-ORFA.

To generate a *actVI-orfA*^-^ mutant, plasmid pXZ14 (Fig 2) was constructed through removal of the *actVI-orfA* gene from pXZ11, which was introduced into *S*. *coelicolor* M1152 to generate the transformant strain M1152::pXZ14. For metabolism analysis, the abovementioned three strains were grown in R4 media in parallel. The metabolites were extracted, sampled in time-course, and analyzed with HPLC. As the production of secondary metabolites was to be investigated, to minimize the difference in growth phases between cultures, the initial cell densities were normalized (see Metabolism analysis in Materials and methods).

The production of **4** in M1152::pXZ14 was validated by comparing its metabolism profile to those of M1152::pXZ4 and M1152::pXZ11 (Fig 3A), which reinforced previous suggestion that ActVI-ORFA protein is not required for the pyran ring cyclization [13]. In addition, an unknown metabolite(s) with a featured UV-visible absorbance at 334 nm (shown in S1 Fig) was detected in the culture medium of M1152::pXZ14, although it is noteworthy that the metabolite(s) was also present in trace quantities in extracts from M1152::pXZ11 (Fig 3A).

### Structure elucidation of two new compounds 5 and 6

In order to elucidate the structure of the compound, the metabolite(s) was isolated from a large-scale culture of M1152::pXZ14 for analysis by HRMS (S1 Fig) and NMR spectrometry. We show the chemical shifts and *J*-coupling data in Table 1, the raw NMR spectra in S3–S7 Figs, and selected NMR spectra showing the structural features of the compounds in S8C Fig. Compound **5** ([M-H]^-^, ESI^-^ obs. 321.0965, calc. 321.0974) was found to be unstable and gradually degraded to **6** ([M-H]^-^, ESI^-^ obs. 303.0860, calc. 303.0869) during the characterization with an initial 10:1 ratio based on proton NMR measurements. The ratio of **5** to **6** changed to 1:0.5 in 5 days and the next time when the sample was measured after 41 days, the ratio further changed to 1:1 (S2 Fig). Our chemical characterization revealed that **6** is the deoxy form of **7** that has previously been isolated from cultures of *S*. *coelicolor*.

**Table 1:** Chemical shifts and *J*-coupling data of compounds 5 and 6.

Position	5		6	
	^13^C	^1^H	^13^C	^1^H
	δ ppm	δ ppm, *J* Hz	δ ppm	δ ppm, *J* Hz
**1**	171.5	exchange	171.5	exchange
**2**	40.0^a^	2.62 dd, 5.9; 15.6;	38.1	2.50^a^ m
		2.48 dd, m^b^		2.47 m^b^
**3**	67.0	5.34 m	70.8	5.31 m
**4**	44.6	1.93 dd, 8.0; ND^b^	39.5^a^	2.88 m, 2H
		1.80 dd, 4.2; 14.9;		
**5**	71.1	exchange	135.8	
**6**	40.4	2.95 dd, 1.8; 16.4;	118.2	7.00 d, 0.8
		3.07 d, 16.4		
**7**	143.2		136.5	
**8**	120.0	6.78 dd, 0.7; 7.8;	117.6	7.13 dd, 0.7; 8.3;
**9**	136.5	7.45 dd, 7.8; 8.4;	127.1	7.20 dd, 7.6; 8.3;
**10**	114.7	6.76 dd, 8.4; 0.7	107.7	6.60 dd, 0.7; 7.6;
**11**	161.3	12.23 s	155.3	exchange
**12**	116.2		113.9	
**13**	204.6		155.3	
**14**	50.8	2.90 d, 16.7	109.5	6.53 d, 0.8
		2.68 dd, 1.8; 16.7;		
**15**	169.5		169.6	
**16**	21.1	1.97 s, 3H	20.8	1.94 s, 3H

^a^The signal is under the solvent peak, data obtained from HSQC and HMBC

^b^The signal is overlapping with other signals

The NMR spectra indicated two similar compounds, which were distinguished based on their initial difference in ratios (described above) and separate correlations in the 2D spectra. The main compound **5** was found to be a two-ringed structure with a single aromatic ring. The key to the elucidation of the structure of **5** was the presence of the aromatic ring with three protons in a single spin system corresponding to H8–H10 (S8Ci Fig, referring to the atom numbering in Fig 1). The second ring system was not aromatic but contained a ketone at C13 (δ 204.6) and a hydroxyl group at C5 (δ 71.1) based on carbon shifts and by HMBC correlation from H6, OH11, and H14 to C13 and from H3, H4, H6, and H14 to C5 (shown in S8B and S8Cii–S8Civ Fig). Furthermore, the shifts for C6 (δ 40.4) and C14 (δ 50.8) and the corresponding protons H6 (δ 2.95 and 3.07) and H14 (δ 2.68 and 2.90) indicate methylenes in an aliphatic ring structure.

In turn, compound **6** seemingly resulted from **5** via a dehydration reaction and contained two aromatic rings. In comparison to **5**, the ketone at C13 (δ 204.6 *vs*. 155.3) converted to a hydroxyl through enolization, and the hydroxyl at C5 (δ 71.1 *vs*. 135.8) was lost together with H6 to create the second aromatic ring. The carbon shift for C6 in **5** (δ 40.4) indicates a methylene carbon, whereas C6 in **6** (δ 118.2) corresponds to an aromatic carbon, as is the case similarly with C14 (δ 50.8 *vs*. 109.5). These differences can also be seen in the proton (S3 Fig) and the HSQC (S6 and S8Ci Figs) spectra, where the total five aromatic protons in **6** are shown. Specifically, three of the aromatic protons in **6**, H8–H10, have similar coupling constants as in **5** (*J*_8,9_ = 8.3, *J*_9,10_ = 7.6, and *J*_8,10_ = 0.7), while the two additional aromatic protons, H6 and H14, both have the same small coupling constant (*J =* 0.8 Hz) that indicates the molecular symmetry.

### Dynamic metabolism analysis reveals that *actVI-orfA* prevents formation of shunt metabolites

After identifying the unknown metabolites **5**/**6**, we analyzed dynamic metabolism profiles of M1152::pXZ11 and M1152::pXZ14. Since the yield of a single compound and even the metabolism profiles may vary between different individual clones, we used the yield ratio of **4** to **5**/**6** as the metric rather than the absolute yields of the metabolites. The time-course analysis presented in Fig 3B demonstrates that from day 4, the ratio for the *actVI-orfA*^-^ mutant M1152::pXZ14 were significantly lower than that for M1152::pXZ11. The trend continued on day 6 and day 8 until the end of the cultivation period. This result implies that ActVI-ORFA plays a role in limiting the generation of the shunt metabolites **5**/**6**.

### Molecular docking guided site-directed mutagenesis of ActVI-ORFA

Based on our proposed biosynthetic model (Fig 1), the spontaneous conversion from **3a** to **3b** is the essential step leading to the production of the shunt metabolites **5**/**6**. Accordingly, we surmised that if ActVI-ORFA functions to limit the production of the shunt metabolites, it would need to interact with **3b** and protect this molecule from converting to **5**. However, the instability of **3b** prevented experimental work and therefore we focused on computational docking modeling to test the hypothesis.

In the previous structural analysis [27], a solvent-accessible cavity was revealed in ActVI-ORFA and in a homologous protein of unknown function Caci_6494. Residues surrounding the cavity in ActVI-ORFA and Caci_6494 align well, with only the side chains of two residues, H45 and R136, orienting differently. The co-crystal structure of Caci_6494 in complex with **4** in the active site cavity has been obtained. Although **4** is not the correct ligand of Caci_6494, hydrogen-bonding interactions between the side chains of residues Y33 and R76 and the ligand were observed [27].

**Fig 4:**
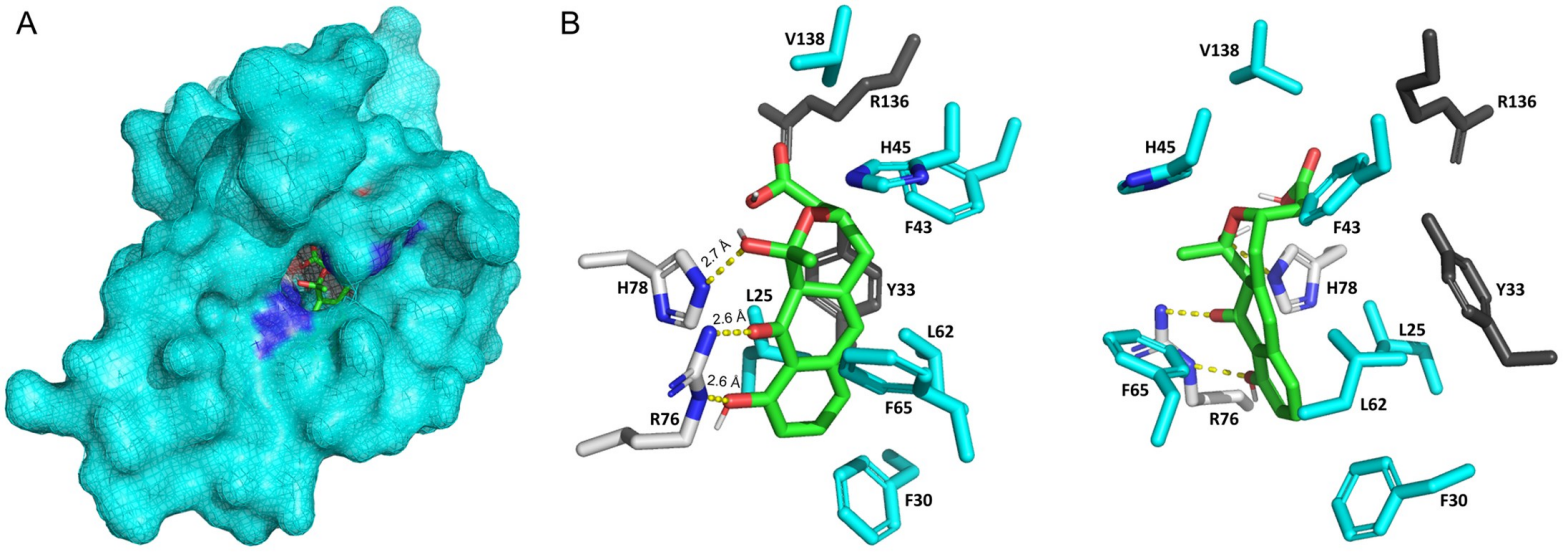
Molecular docking analysis of ActVI-ORFA with 3b. (A) The positioning of **3b** within the solvent-accessible cavity of ActVI-ORFA, where blue and red surface shading represent regions of positive and negative electrostatic potential, respectively. (B) The residues of ActVI-ORFA interacting with **3b**, with hydrogen bonds illustrated by yellow dashed lines and the measured distances between the interacting atoms, in Angstroms (Å), are labeled (left). Residues Y33 and R136 are shown in dark grey. An additional view of the active site, rotated around the y-axis by 270°, shows the positions of Y33 and R136 in another angle (right).

Our docking analysis (Fig 4) identified three hydrogen bonds between the side chains of R76 and H78 and the atoms/groups OH11, O13, and OH15 of **3b** (atom numbering based on Fig 1). Y33 was not identified as a residue interacting with the ligand in the model. R136 was not suggested to have a polar interaction with **3b**, whereas L25, F30, F43, H45, L62, F65, and V138 were in the radius of van der Waals contacts (Fig 4B). The two residues participating in hydrogen-bonding interactions with the ligand in our docking model, R76 and H78, were selected for substitution with alanine. Although they were not identified in the modeling, Y33, whose counterpart forms a direct hydrogen bond with **4** in the crystal structure of Caci_6494, and R136, which orients differently in the solvent-accessible cavity in ActVI-ORFA and Caci_6494, were selected to be substituted as well.

Next, we synthesized and cloned the wild type *actVI-orfA* gene and the variants, R76A, H78A, Y33A, and R136A, into the *actVI-orfA*^-^ plasmid pXZ14 to generate plasmids pRW_wt, pRW_R76A, pRW_H78A, pRW_Y33A, and pRW_R136A, respectively. These plasmids were introduced into the host *S*. *coelicolor* M1152. The metabolism profiles of the resultant transformant strains were analyzed to investigate the effects of the mutations.

**Fig 5:**
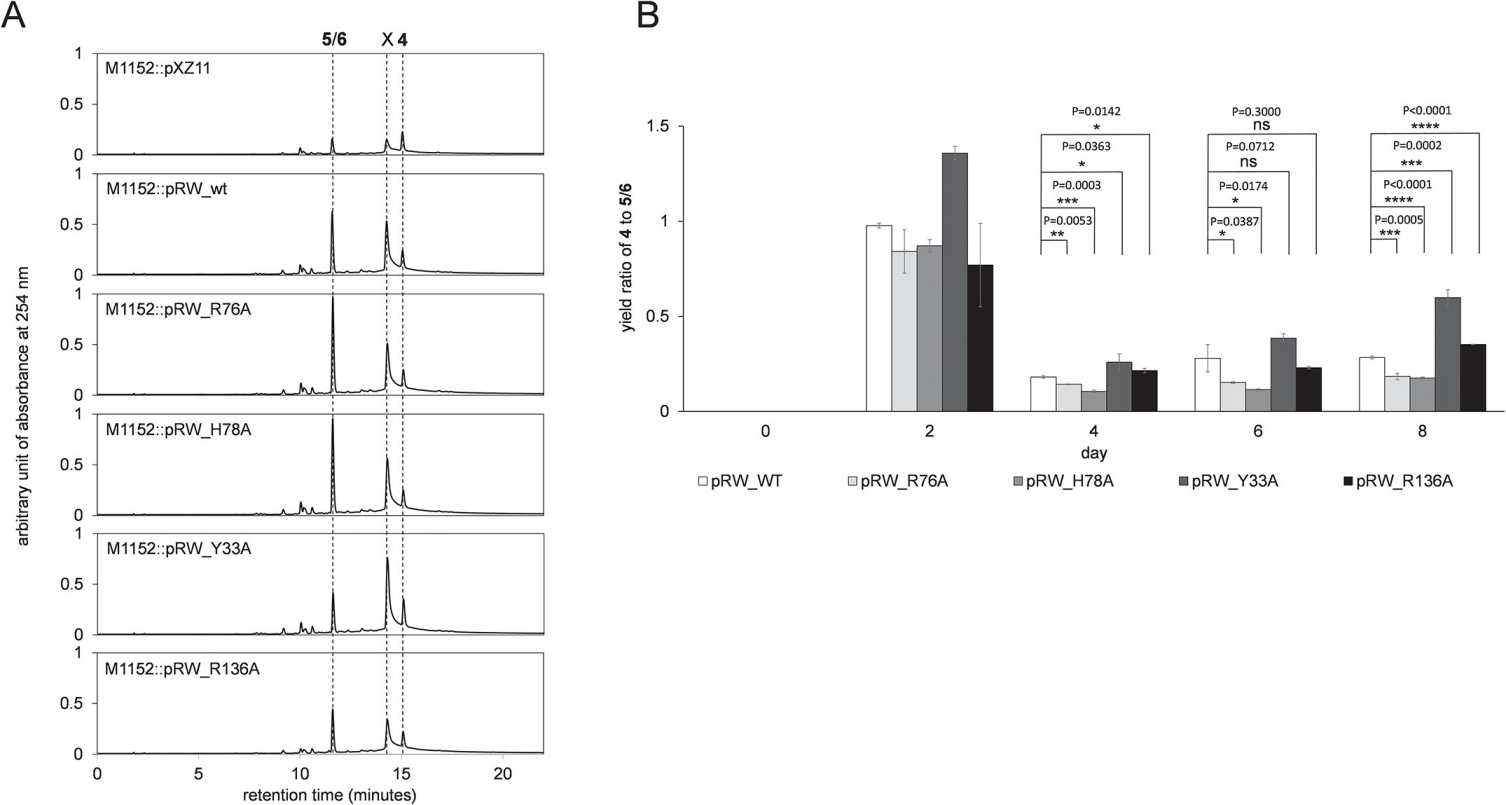
The metabolism analysis of *actVI-orfA* variants. (A) The metabolic profiles of transformant strains M1152::pXZ11 (as the reference), M1152::pRW_wt, M1152::pRW_R76A, M1152::pRW_H78A, M1152::pRW_Y33A, and M1152::pRW_R136A on day 8. Peaks of **4**, **5**/**6**, and compound X are labeled. The retention times (minutes) of the metabolites are: **5**/**6** = 11.59, **4** = 15.03, and X = 14.26. (B) The yield ratios of **4** to **5**/**6** in *actVI-orfA* mutant strains. Peak areas at 254 nm in HPLC spectra are used to represent the yields. Error bars represent the standard deviations (n = 3). The significant differences are evaluated with unpaired student t-test and labeled with ns (not significant), * (P≤ 0.05), ** (P ≤ 0.01), *** (P ≤ 0.001), and **** (P ≤ 0.0001) along with the calculated P values.

The production of **4** and **5**/**6** in the transformant strains was verified by comparing with the metabolism profile of M1152::pXZ11 that was grown in parallel (Fig 5A). Interestingly, a new metabolite, labeled as X, was observed in addition to **4** and **5**/**6** in the HPLC traces of all tested strains. This metabolite displayed UV-Vis absorbances at 219 nm, 288 nm, and 376 nm (S9 Fig), which are not consistent with the spectrum of any known pathway intermediate. In order to determine if X was related to (*S*)-DNPA biosynthesis, we isolated the compound from the culture of M1152::pRW_Y33A for analysis by HRMS. The mass spectrum ([M-H]^-^, ESI^-^) showed a mass of 325.18288 (S9 Fig), consistent with the molecular formula C_11_H_22_N_10_O_2_ or C_10_H_26_N_6_O_6_, instead of a 16-carbon compound. For these reasons, the metabolite, which was highly unstable and could not be isolated for NMR analysis, did not appear to be related to actinorhodin biosynthesis.

We carried out another time series to acquire dynamic metabolism profiles of the pRW transformant strains and analyzed the yield ratios of **4** to **5**/**6** (Fig 5B). From day 4, the ratios for M1152::pRW_R76A and M1152::pRW_H78A were significantly lower than those for M1152::pRW_wt. This result is consistent with the docking analysis, indicating the importance of R76 and H78 in the function of ActVI-ORFA. Also, it suggested that this function is carried out by interacting with **3b**, which leads to the formation of **5** (refer to Fig 1). In contrast, M1152::pRW_Y33A and M1152::pRW_R136A did not show a significant difference in the yield ratios from M1152::pRW_wt stably (Fig 5B). Interestingly, the alanine substitution at these two positions increased the yield ratio of **4** to **5**/**6**, rather than decreasing it. Given that Y33 and R136 did not interact with **3b** in the modeling, the impact on the metabolism may result from a conformational change of the solvent-accessible cavity where the ligand binds. Further investigation is required to answer this question. However, the results from the test of variants Y33A and R136A indicated that the alanine substitutions in the active site did not disrupt the folding of ActVI-ORFA, which would have resulted in differences in metabolism profiles (similar to the strains lacking *actVI-orfA*), thus these two mutations likely affected the protein-ligand interactions.

## Discussion

The role of ActVI-ORFA in the biosynthesis of actinorhodin has been controversial and it has been suggested to function as a cyclase, an accessory protein, or a transcriptional regulator. One of the challenges in investigating the functions of proteins involved in early steps of PK-II biosynthesis is the instability of pathway intermediates, which has excluded the use of biochemical assays. On the other hand, metabolism analyses performed *in vivo* may be subject to interference from endogenous secondary metabolism of the host. The previous functional analyses of ActVI-ORFA were performed using *actVI-orfA*^-^ mutants derived from the wild type *S*. *coelicolor* [24–26]. Here, in contrast, we used a host from which four competing secondary metabolism pathways have been removed and a “bottom-up” assembly of the actinorhodin pathway to test the function of ActVI-ORFA. Furthermore, we devised methods to control initial cell density in *Streptomyces* cultures by fragmentation of mycelium and carried out careful time-course analysis of metabolite production to be able to perform comparative dynamic metabolism analysis of *actVI-orfA* strains.

The results of our analyses suggested a metabolic flux director function for ActVI-ORFA, which would bind and protect the unstable intermediate **3b** from degradation and direct carbon flux towards actinorhodin through the spontaneous isomerization between **3a** and **3b**. Our data demonstrate that addition of *actVI-orfA* to constructs with early actinorhodin biosynthesis genes prevents formation of the shunt metabolites **5**/**6** newly identified in this study. We propose a metabolic model, where **5** may be formed from **3b** via cleavage of the C14-C15 bond. The stereospecificity of C3 originating in the reaction from **1** to **2** will be eliminated when converting to **5**, and the C5 of **5** can be a potential stereocenter. However, as the key focus is on the functional characterization of ActVI-ORFA, while interesting, the optical rotation of compound **5** is not measured in this study. Dehydration of **5** in the aqueous environment of the culture medium may lead to formation of **6**. Alternatively, under oxidative conditions, **6** may also be converted to **7**.

In order to gain more evidence for our functional proposal for ActVI-ORFA, we performed molecular docking analysis to model **3b** in the active site. Compound **3b** is a highly amphipathic molecule with a polycyclic ring system that has a hydrophobic and more hydrophilic side. The analysis revealed that **3b** may be positioned in the active site in a suitable conformation to accommodate these features. On one side, the active site cavity has a hydrophobic pocket shaped by five amino acid residues, while the other side allows formation of three hydrogens bonds between the **3b** ligand and the amino acids R76 and H78. The hydrogen bonds involve OH15 and O13 of **3b**, where the retro-Claisen type reaction could have occurred to result in the cleavage of the carbon-carbon bond between C14 and C15. Our site-directed mutagenesis results revealed that mutation of R76 or H78 to Ala is detrimental to the activity of ActVI-ORFA and the metabolic profiles demonstrate an increase in formation of the shunt metabolites **5**/**6**.

The biosynthesis of PK-II compounds is initiated with the formation of a highly reactive polyketide carbon chain, which needs to be folded in a controlled manner by a cascade of multiple enzymatic reactions to produce stable compounds. Overproductions of shunt metabolites consume resources of the host cell and therefore it is plausible that such type of proteins are common on PK-II pathways to direct carbon flux towards desired end products. In effect, we noted that ActVI-ORFA homologs are encoded by genes in the biosynthetic gene clusters of many known PK-IIs belonging to different structural subclasses, such as *aln2* [22], *med-orf10* [34], *gra-orf31* [35], and *fren-orfX* [36] in clusters of alnumycin, medermycin, granaticin, and frenolicin of the benzoisochromanequinone (BIQ) subclass; *dpsH* [20], *snoO* [37], *chaN* [38], *ara-orf8* [39], *nivV1* and *nivE1* [40], *aknV* [41], and *stfX* [42] in clusters of daunorubicin, nogalamycin, chartreusin, aranciamycin, nivetetracyclates, aclacinomycin, and steffimycin in the anthracycline (ANT) subclass; and *ctcH* [43], *oxyI* [44], *pokC1* [45], *mtmX* [46], and *dacG* [47] in clusters of chlortetracycline, oxytetracycline, polyketomycin, mithramycin, and dactylocycline belonging to the tetracycline (TET) subclass. These proteins possess amino acid sequence identity to ActVI-ORFA from 28% to 57%. The functions of all these proteins are currently unclear, and future studies are required to reveal if the existence of metabolic flux directing proteins is a more common phenomenon on PK-II biosynthetic pathways.

## Supporting information

S1 Appendix Supplementary information about materials, detailed procedures, DNA sequences, and plasmid maps. (DOCX)

S1 Fig UV-vis spectrum (top) and HRMS (ESI) in negative ionization mode (bottom) of 5/6. The featured absorbance wavelengths (nm) are labeled. HRMS (ESI) m/z for **5** [M-H]^-^: observed: 321.0965, calculated: 321.0974; for **6** [M-H]^-^: observed: 303.0860, calculated: 303.0869.
(PDF)

S2 Fig ^1^H spectrum measured on different days indicating the spontaneous conversion from 5 to 6. (PDF)

S3 Fig ^1^H spectrum of 5/6 measured at 600 MHz and 5:6 = 10:1. (PDF)

S4 Fig ^13^C spectrum of 5/6 at 5:6 = 1:1. (PDF)

S5 Fig COSY spectrum of 5/6 at 5:6 = 1:0.5. (PDF)

S6 Fig HSQC spectrum of 5/6 at 5:6 = 1:0.5. (PDF)

S7 Fig HMBC spectrum of 5/6 at 5:6 = 1:0.5. (PDF)

S8 Fig NMR characterization of 5/6. (A) Molecular structures and numberings of **5** and **6**; (B) HMBC correlations in the compounds. Two-colored lines, red and blue, are used to show the many correlations clearly; (C) Relevant features of the compound are shown in the selected spectra. The atoms and the compounds **5** or **6** are labeled. i) HSQC spectrum indicating the aromatic hydrogens in both compounds. Compound **5** has three aromatic hydrogens linked to carbons, all coupled. Compound **6** has five aromatic hydrogens linked to carbons, of which three are visibly coupled (*J* = 0.7, 7.6, 8.3), and a coupling between the other two was indicated (*J* = 0.8); ii) HMBC spectrum showing the correlations of H3 (left for **5** and right for **6**). These signals show the major differences between these two compounds. Both compounds have correlations to C1, C2, C4, C5, and C15, but the shifts are different for C5 and C4 (δ 71.1 *vs*. δ 135.8, δ 44.6 *vs*. δ 39.5, respectively). The C5 shift in **5** indicates an attached OH-group, whereas the shift δ 135.8 for **6** corresponds to an aromatic quaternary carbon. The change of the C4 shift is due to the structural difference at C5; iii) and iv) HMBC spectra showing the correlations of H6 and C13 and OH11 and C13 in **5**, respectively; v) HSQC spectrum showing the correlations of H2 to C2 in **5** (δ 2.62 and 2.48 to 40.0) and **6** (δ 2.50 and 2.47 to 38.1), which are under the solvent peaks; vi) The carbon peak of C13 (δ 204.6) in **5**. In that region, only the peak for **5** is visible. No corresponding peak is observed for **6**.
(PDF)

S9 Fig UV-vis spectrum (top) and HRMS (ESI) in negative ionization mode (bottom) of compound X. The featured absorbance wavelengths (nm) are labeled. Observed HRMS ([M-H]^-^, ESI) m/z: 325.18228. Predicted molecular formula: C_11_H_22_N_10_O_2_ and C_10_H_26_N_6_O_6_.
(PDF)

S1 Data (XLSX)
