# Supplementary material for: ActVI-ORFA directs metabolic flux towards actinorhodin by preventing intermediate degradation
Source: PLoS One. 2024 Aug 9;19(8):e0308684. doi: 10.1371/journal.pone.0308684 (PMC11315284; doi:10.1371/journal.pone.0308684)
Supplement: S1 Appendix — (DOCX) [file pone.0308684.s001.docx]

## **Materials**

General biochemical reagents and organic solvents were purchased from VWR (Atlanta, GA USA) and Sigma-Aldrich (Darmstadt, Germany) and were used without further purification. All bacterial culture media were made with DI water. Ampicillin sodium salt was from USB Corporation (Cleveland, OH USA) or Sigma-Aldrich (Darmstadt, Germany). Apramycin sulfate salt was from Sigma-Aldrich at St. Louis, MO USA or at Darmstadt, Germany. UltraClean Microbial DNA Isolation Kit from MO BIO Laboratories, Inc. (Carlsbad, CA USA) was used for the genomic DNA extraction. Restriction enzymes, T4 DNA ligase, and calf intestinal alkaline phosphatase (CIP) used in cloning were purchased from New England Biolabs (Ipswich, MA USA) or Thermo Fisher Scientific Inc. (Waltham, MA USA). For cloning, DNA was extracted from agarose gel with Gel DNA Recovery Kit from Zymo Research (Irvine, CA USA) or GeneJET Gel Extraction Kit from Thermo Fisher Scientific Inc. (Waltham, MA USA) and was concentrated with DNA Clean & Concentrator Kit from Zymo Research (Irvine, CA USA). Plasmids were extracted with QIAprep Spin Miniprep Kit from QIAGEN (Valencia, CA USA) or GeneJET Plasmid Miniprep Kit from Thermo Fisher Scientific Inc. (Waltham, MA USA). DNA concentrations were determined with a Nanodrop 2000 UV-vis spectrophotometer (Thermo Scientific). Plasmids were sequenced at Genewiz (South Plainfield, NJ) or at Eurofins Genomics (Ebersberg, Germany). The unpaired Student’s t-tests were performed with the software GraphPad Prism by Dotmatics.

## **Bacterial culture and transformation**

For genomic DNA extraction, *S. coelicolor* A3(2) was grown in GYM medium at 28^o^C, 250 rpm to the stationary phase. Two mL of the culture medium were sampled for DNA extraction.

For cloning, *E. coli* DH5α and *E. coli* ET12567 competent cells were prepared using the rubidium chloride method [1]. Standard protocols [1] were followed in *E. coli* transformation. *E. coli* DH5α transformants were selected on LB agar plates containing 100 μg/mL ampicillin or 50 μg/mL apramycin at 37^o^C overnight (12-16 h). The resultant single colonies were grown in 4 mL of LB broth at 37^o^C, 250 rpm overnight (12-16 h). Cells were collected by centrifugation for plasmid extraction. Regular plasmids were transformed into *E. coli* ET12567 to produce the non-methylated plasmid DNA for *Streptomyces* transformation. The resultant transformant colonies were grown in 15 mL of LB broth at 37^o^C, 250 rpm overnight (12-16 h) for plasmid extraction.

Both protoplast transformation and conjugative transfer were used to introduce the non-methylated plasmid DNA into *S. coelicolor* M1152. For preparing the protoplasts, *S. coelicolor* M1152 was grown in YEME medium containing 0.5% glycine. All the other steps followed the standard protocol [2].

Except for the R5 media culture, in all *Streptomyces* culture, autoclaved glass beads (4 mm) were placed in the flasks to break down the cell chunks forming during the cultivation.

## **Sequences of primers and synthesized genes and maps of plasmids**

Sequences of the synthesized *actVI-orfA* variant genes are shown below. Restriction sites for cloning are in Italic. The site-directed mutagenesis is highlighted with the Bold font.

*actVI-orfA*_wt:

*TCTAGA*GGCGACGAGCCTGGCAGCGCCCACTCGAACCCGGCTCGAGGGAACTTCGAGGCCACCGGGCGACCGTGGCCGTATGACCATCACCGCGCTTCCCACCGGCCTGTACGCCGAGGTCCTGTCGTTCTACGGCCACCAGATGCAGAAGCTGGACGGCCGTGACTTCGCCGGCTACGCCGCGACCTTCACCGAGGACGGCGAGTTCAGGCACTCGCCCTCGCTGCCTGCCGCCCACACCCGTGCCGGGATCACCGCCGTCCTGGAGGACTTCCACCGCAAGTTCGACGCCCGCAAAATCCAGCGCAGGCACTGGTTCGACCACACGGCGCTGAGCCAGGCGTCCGACGGCTCGATCACGGCGACCAGCTACTGCCTGGTGCTCACCGTCCACGCCGACGTCAAGGCGCCGGAGTTCGGGCCGAGTTGCCTCGTGCACGACGTCCTGGTCCGGGGGGCGGACGGCGAGTTGCTGCTGCGCTCCCGCCACGTCACCCATGACCACGTCTTCCCGGCCTGA*AAGCTT*

*actVI-orfA*_R76A:

*TCTAGA*GGCGACGAGCCTGGCAGCGCCCACTCGAACCCGGCTCGAGGGAACTTCGAGGCCACCGGGCGACCGTGGCCGTATGACCATCACCGCGCTTCCCACCGGCCTGTACGCCGAGGTCCTGTCGTTCTACGGCCACCAGATGCAGAAGCTGGACGGCCGTGACTTCGCCGGCTACGCCGCGACCTTCACCGAGGACGGCGAGTTCAGGCACTCGCCCTCGCTGCCTGCCGCCCACACCCGTGCCGGGATCACCGCCGTCCTGGAGGACTTCCACCGCAAGTTCGACGCCCGCAAAATCCAG**GC**CAGGCACTGGTTCGACCACACGGCGCTGAGCCAGGCGTCCGACGGCTCGATCACGGCGACCAGCTACTGCCTGGTGCTCACCGTCCACGCCGACGTCAAGGCGCCGGAGTTCGGGCCGAGTTGCCTCGTGCACGACGTCCTGGTCCGGGGGGCGGACGGCGAGTTGCTGCTGCGCTCCCGCCACGTCACCCATGACCACGTCTTCCCGGCCTGA*AAGCTT*

*actVI-orfA*_H78A:

*TCTAGA*GGCGACGAGCCTGGCAGCGCCCACTCGAACCCGGCTCGAGGGAACTTCGAGGCCACCGGGCGACCGTGGCCGTATGACCATCACCGCGCTTCCCACCGGCCTGTACGCCGAGGTCCTGTCGTTCTACGGCCACCAGATGCAGAAGCTGGACGGCCGTGACTTCGCCGGCTACGCCGCGACCTTCACCGAGGACGGCGAGTTCAGGCACTCGCCCTCGCTGCCTGCCGCCCACACCCGTGCCGGGATCACCGCCGTCCTGGAGGACTTCCACCGCAAGTTCGACGCCCGCAAAATCCAGCGCAGG**GC**CTGGTTCGACCACACGGCGCTGAGCCAGGCGTCCGACGGCTCGATCACGGCGACCAGCTACTGCCTGGTGCTCACCGTCCACGCCGACGTCAAGGCGCCGGAGTTCGGGCCGAGTTGCCTCGTGCACGACGTCCTGGTCCGGGGGGCGGACGGCGAGTTGCTGCTGCGCTCCCGCCACGTCACCCATGACCACGTCTTCCCGGCCTGA*AAGCTT*

*actVI-orfA*_Y33A:

*TCTAGA*GGCGACGAGCCTGGCAGCGCCCACTCGAACCCGGCTCGAGGGAACTTCGAGGCCACCGGGCGACCGTGGCCGTATGACCATCACCGCGCTTCCCACCGGCCTGTACGCCGAGGTCCTGTCGTTCTACGGCCACCAGATGCAGAAGCTGGACGGCCGTGACTTCGCCGGC**GC**CGCCGCGACCTTCACCGAGGACGGCGAGTTCAGGCACTCGCCCTCGCTGCCTGCCGCCCACACCCGTGCCGGGATCACCGCCGTCCTGGAGGACTTCCACCGCAAGTTCGACGCCCGCAAAATCCAGCGCAGGCACTGGTTCGACCACACGGCGCTGAGCCAGGCGTCCGACGGCTCGATCACGGCGACCAGCTACTGCCTGGTGCTCACCGTCCACGCCGACGTCAAGGCGCCGGAGTTCGGGCCGAGTTGCCTCGTGCACGACGTCCTGGTCCGGGGGGCGGACGGCGAGTTGCTGCTGCGCTCCCGCCACGTCACCCATGACCACGTCTTCCCGGCCTGA*AAGCTT*

*actVI-orfA*_R136A:

*TCTAGA*GGCGACGAGCCTGGCAGCGCCCACTCGAACCCGGCTCGAGGGAACTTCGAGGCCACCGGGCGACCGTGGCCGTATGACCATCACCGCGCTTCCCACCGGCCTGTACGCCGAGGTCCTGTCGTTCTACGGCCACCAGATGCAGAAGCTGGACGGCCGTGACTTCGCCGGCTACGCCGCGACCTTCACCGAGGACGGCGAGTTCAGGCACTCGCCCTCGCTGCCTGCCGCCCACACCCGTGCCGGGATCACCGCCGTCCTGGAGGACTTCCACCGCAAGTTCGACGCCCGCAAAATCCAGCGCAGGCACTGGTTCGACCACACGGCGCTGAGCCAGGCGTCCGACGGCTCGATCACGGCGACCAGCTACTGCCTGGTGCTCACCGTCCACGCCGACGTCAAGGCGCCGGAGTTCGGGCCGAGTTGCCTCGTGCACGACGTCCTGGTCCGGGGGGCGGACGGCGAGTTGCTGCTGCGCTCC**GC**CCACGTCACCCATGACCACGTCTTCCCGGCCTGA*AAGCTT*

The sequences of primers used in cloning are shown below. The priming region is underlined, and the restriction site is in Italic.

| act_KSa_up | TATTTGAA*GAATTC*GAAGGAGCTGTTCGGATTGAAGCGCAG |
| --- | --- |
| act_KSa_dn | TTGATGTAGTCGATGTCCGTCGCGTCCGTG |
| act_KSb_up | ACGGACATCGACTACATCAACGCGCACGGCT |
| act_KSb_dn | ATACCGTGCCGGATGGAGATCTGGCCGGTGT |
| act_ACP_up | TCCATCCGGCACGGTATGCGCGGTCCGAG |
| act_ACP_dn_2 | CGACCAGCTCGTAGAGCCGCCGCGCCG |
| act_Aro_up | GGCTCTACGAGCTGGTCGCACGCGTCGAAGACTG |
| act_Aro_dn | GCCTGCTCGTAGGCGTAGACACCCTCGGCG |
| act_Cyc5_up | TACGCCTACGAGCAGGCCCCCGGCGG |
| act_Cyc5_dn_2 | GCCGTCTAGGCGAGGCAGGTGGGCAGCTGCC |
| act_C3KR_up | GGTTGTTCGCAGAGATCGGAGCGGCC |
| act_C3KR_dn_2 | GGTCATGAG*TCTAGA*TCACTTGGTCTCCTCCTGGGGCTGCTCGGTCCGCTCCAGGGCGG |
| act_C3KR_up_2 | GCCTCGCCTAGACGGCCACGGTCGCCCGGTGGCCTC |
| act_Cyc3_dn | CTCCGATCTCTGCGAACAACCGCTGCTTGAGCG |
| act_colE1_up_2 | GACCAAGTGA*TCTAGA*CTCATGACCAAAATCCCTTAACGTGAG |
| act_ampR_dn_2 | GAACAGCTCCTTC*GAATTC*TTCAAATATGTATCCGCTCATGAGACAATAACCC |

Plasmid maps are shown below. Specifically, plasmids COE-PCR_act_minimal_PKS and COE-PCR_act_Part_1 are produced to assemble the amplicons with COE-PCR; pXZ4 and pXZ11 are constructed in our previous study [3]; pXZ14 and pRW plasmids are constructed in this study.


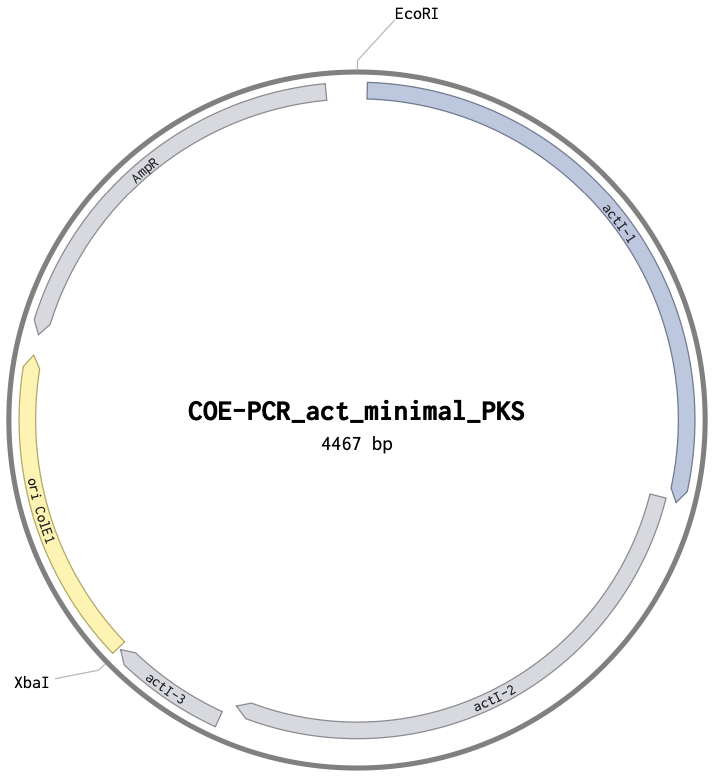

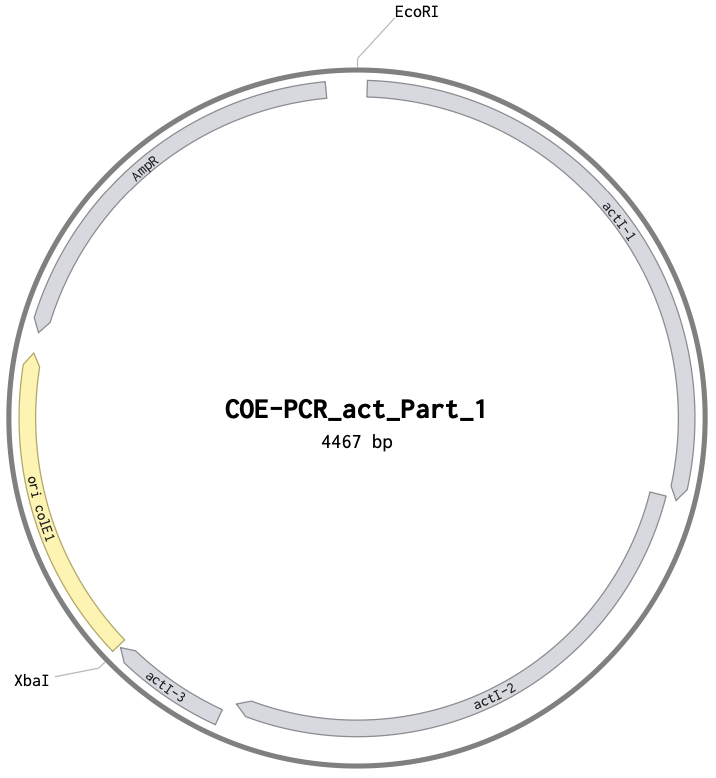


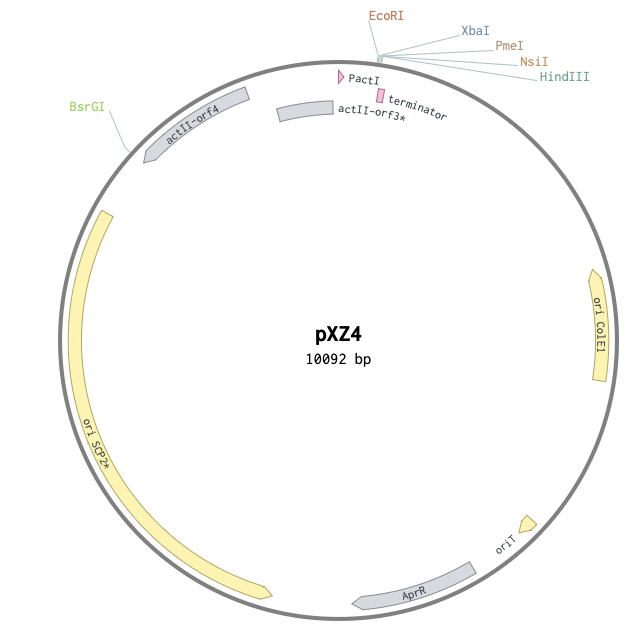

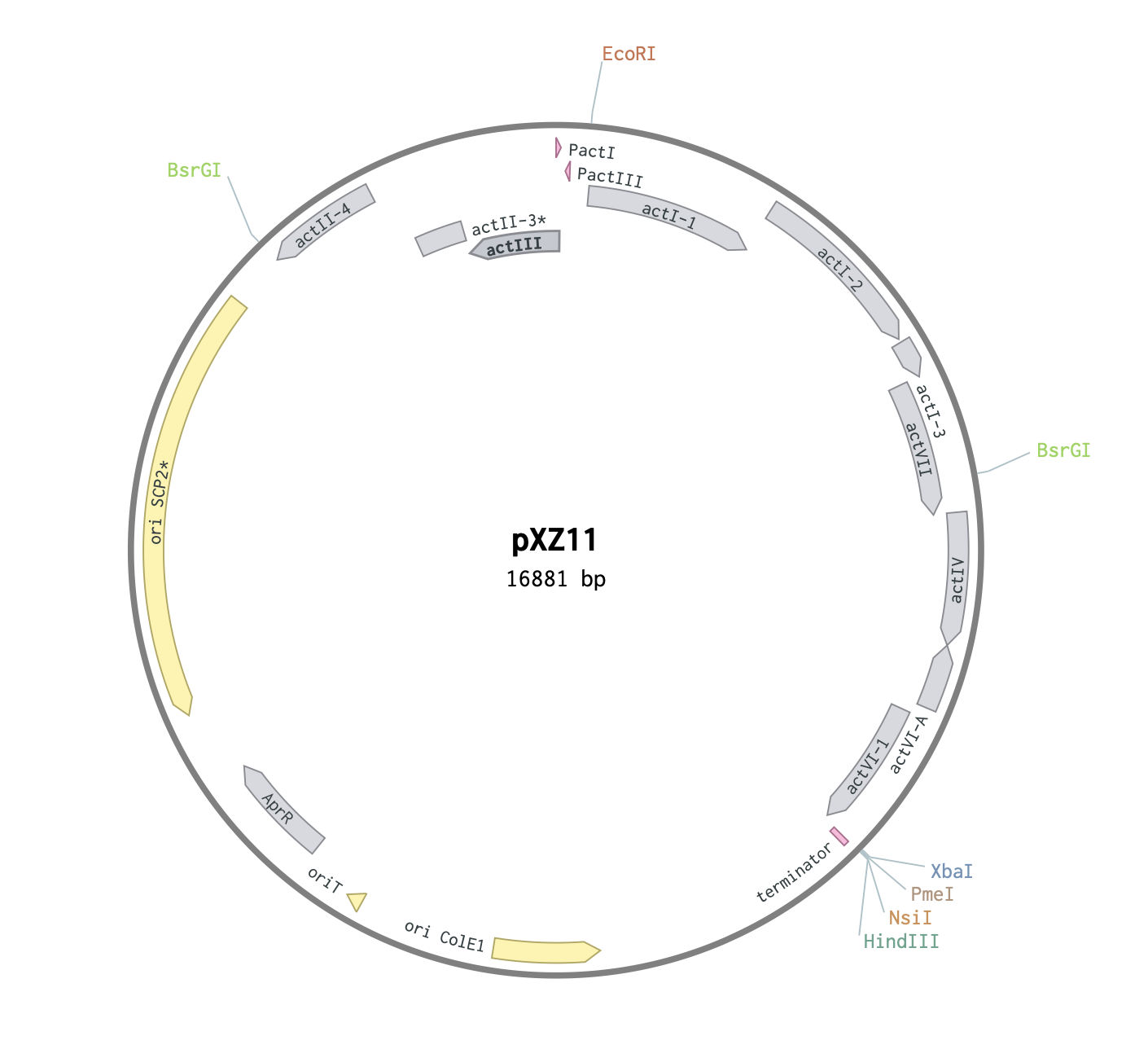


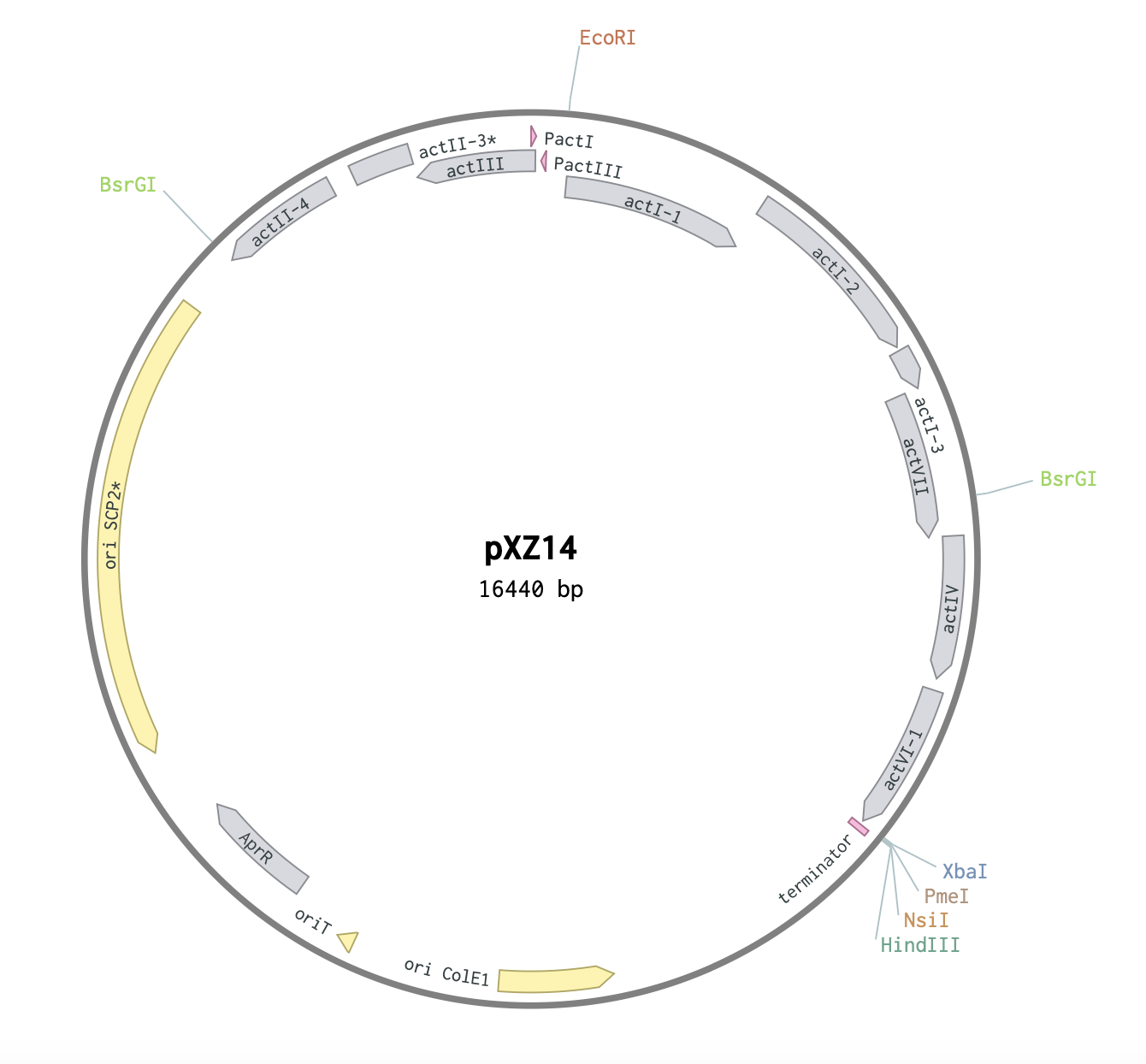

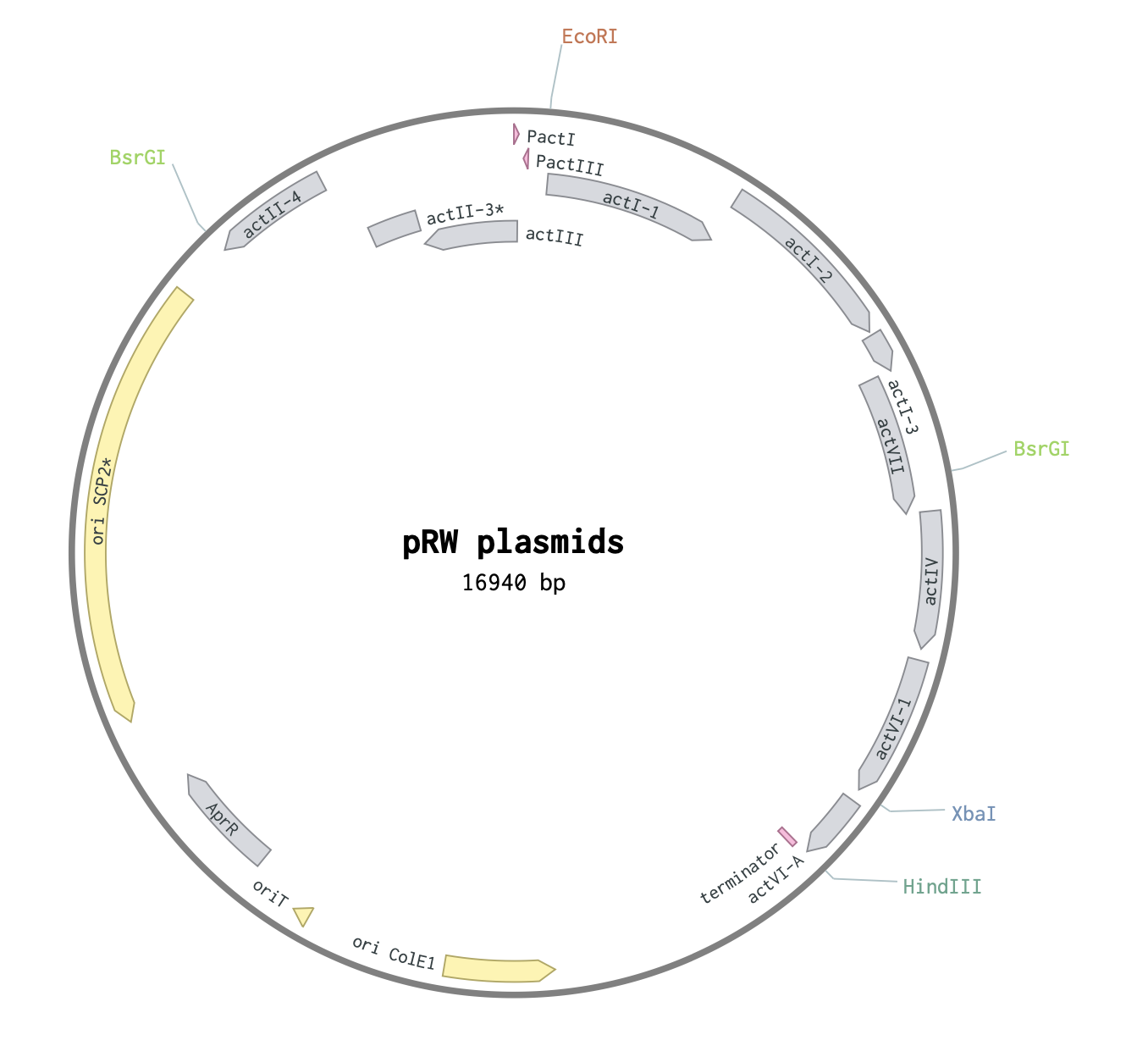


## **NMR data analysis**

Chemical shifts were internally referenced to the solvent signals. The number of hydrogens based on the integrals is one unless otherwise stated. NMR data was analyzed with software Topspin (Bruker Biospin). Signals were annotated with abbreviations below: s, singlet; br, broad; m, multiplet; d, doublet; dd, doublet of doublets; ddd, doublet of doublet of doublets; ND, no data.

## **References**

1. Sambrook J and Russell DW. Molecular cloning: a laboratory manual. 3rd edition. Cold Spring Harbor Press, Cold Spring Harbor, NY; 2001.
2. Kieser T, Bibb MJ, Buttner MJ, Chater KF and Hopwood DA. Practical Streptomyces Genetics. John Innes Foundation, Norwich, U.K.; 2000.
