# Supplementary figures and images for: ActVI-ORFA directs metabolic flux towards actinorhodin by preventing intermediate degradation
Source: PLoS One. 2024 Aug 9;19(8):e0308684. doi: 10.1371/journal.pone.0308684 (PMC11315284; doi:10.1371/journal.pone.0308684)

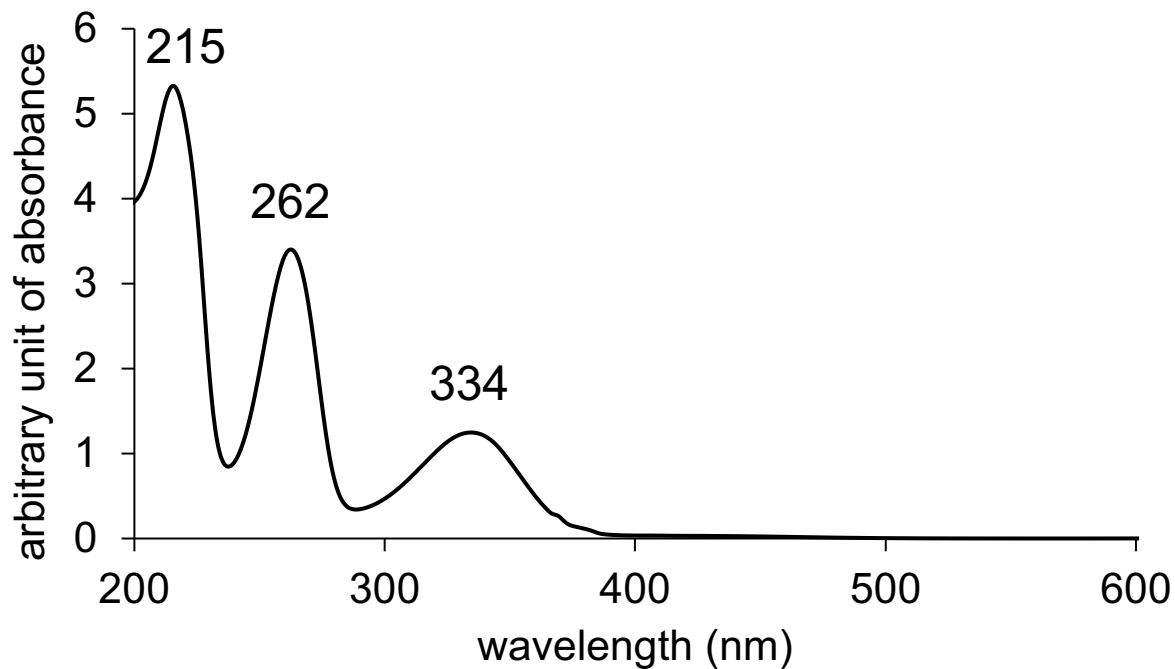

Xuechen Zhu / Melancon XZ-14-P1  
161004\_449 276 (5.629) Cm (201:327-600:700)

UNM MS Facility: LCT Premier  
1: TOF MS ES-  
1.42e4

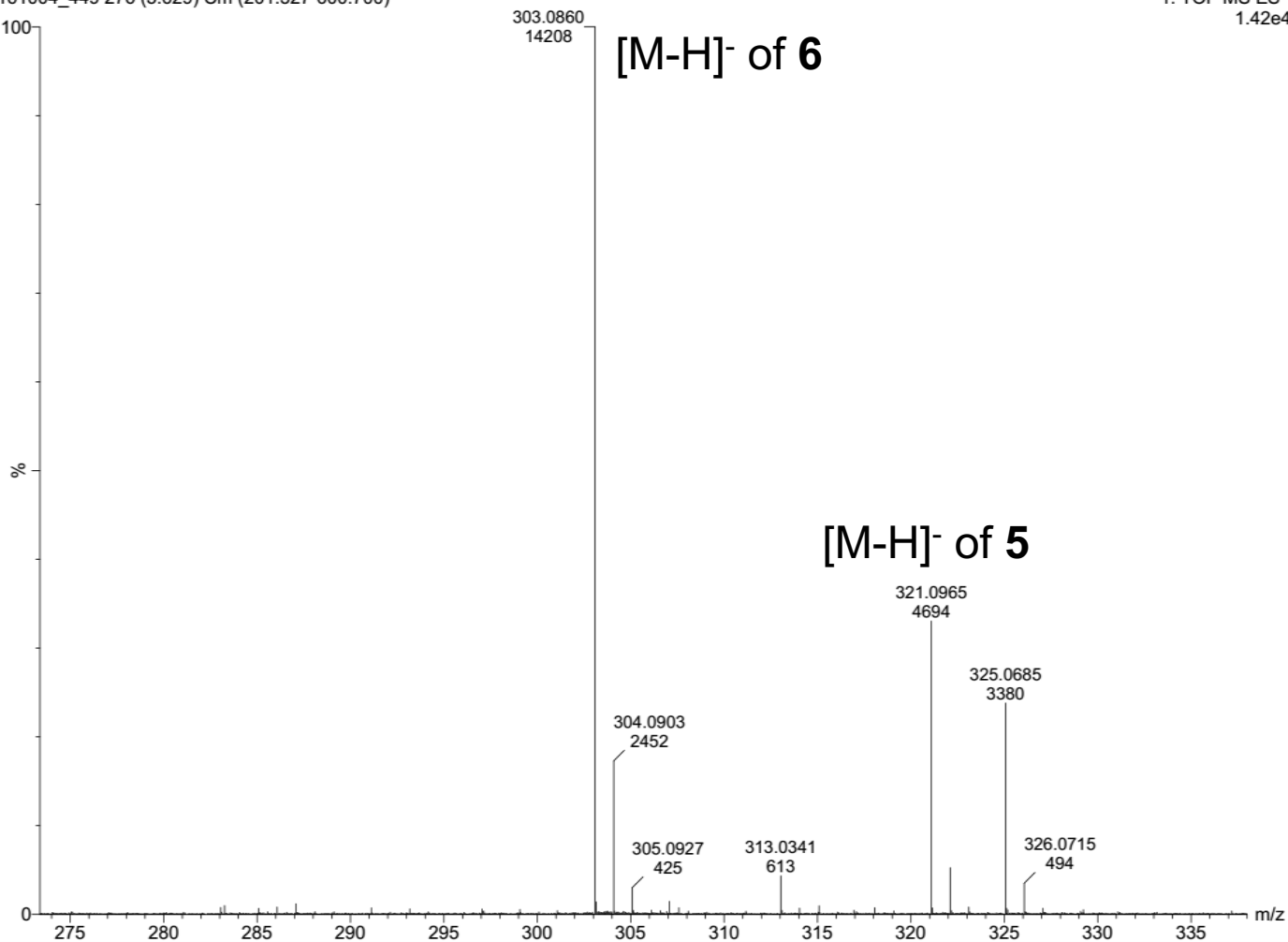

Supplement: S1 Fig — UV-vis spectrum (top) and HRMS (ESI) in negative ionization mode (bottom) of 5/6. The featured absorbance wavelengths (nm) are labeled. HRMS (ESI) m/z for 5 [M-H]-: observed: 321.0965, calculated: 321.0974; for 6 [M-H]-: observed: 303.0860, calculated: 303.0869. (PDF) [file pone.0308684.s002.pdf]

day 41  
5:6 = 1:1  
500 MHz

day 5  
5:6 = 1:0.5  
600 MHz

day 1  
5:6 = 10:1  
600 MHz

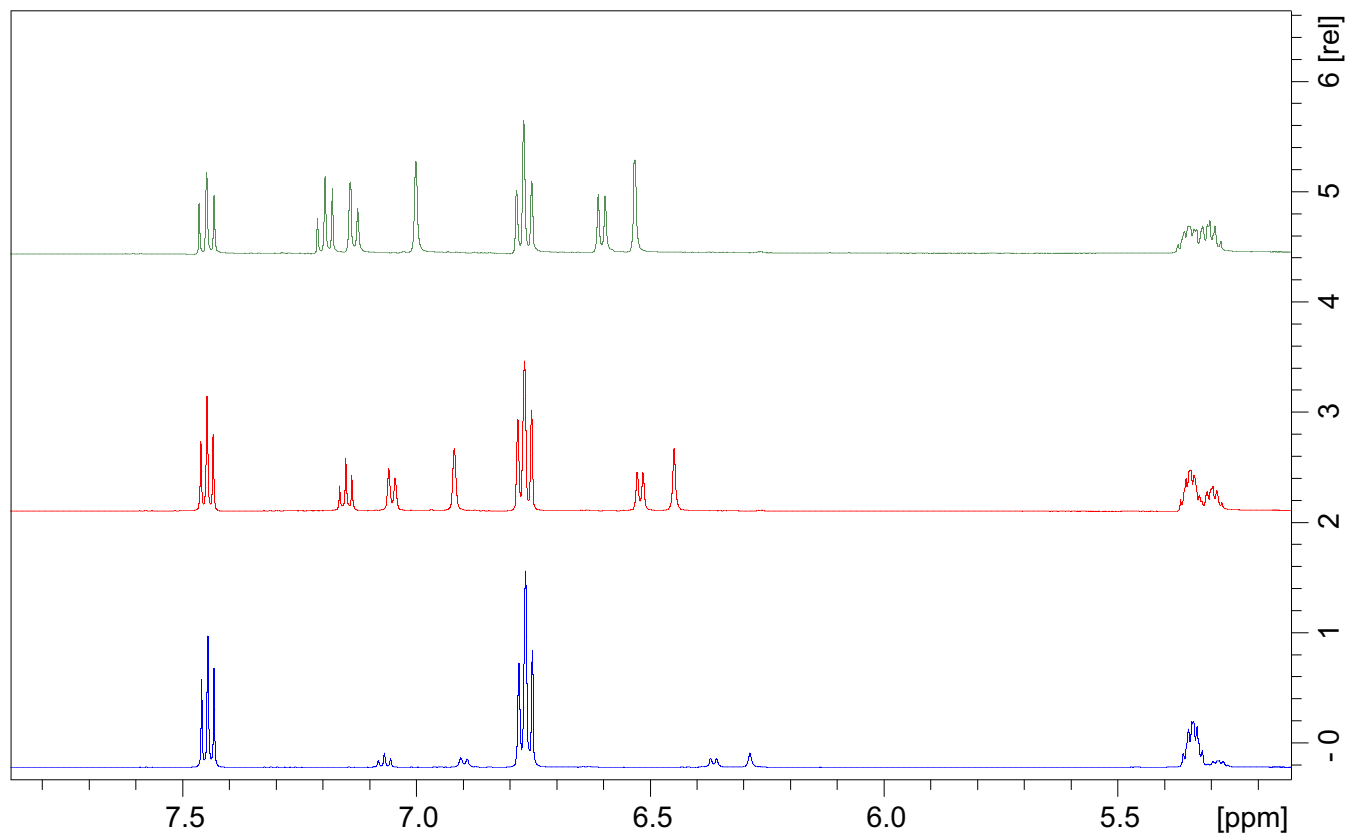

Supplement: S2 Fig — (PDF) [file pone.0308684.s003.pdf]

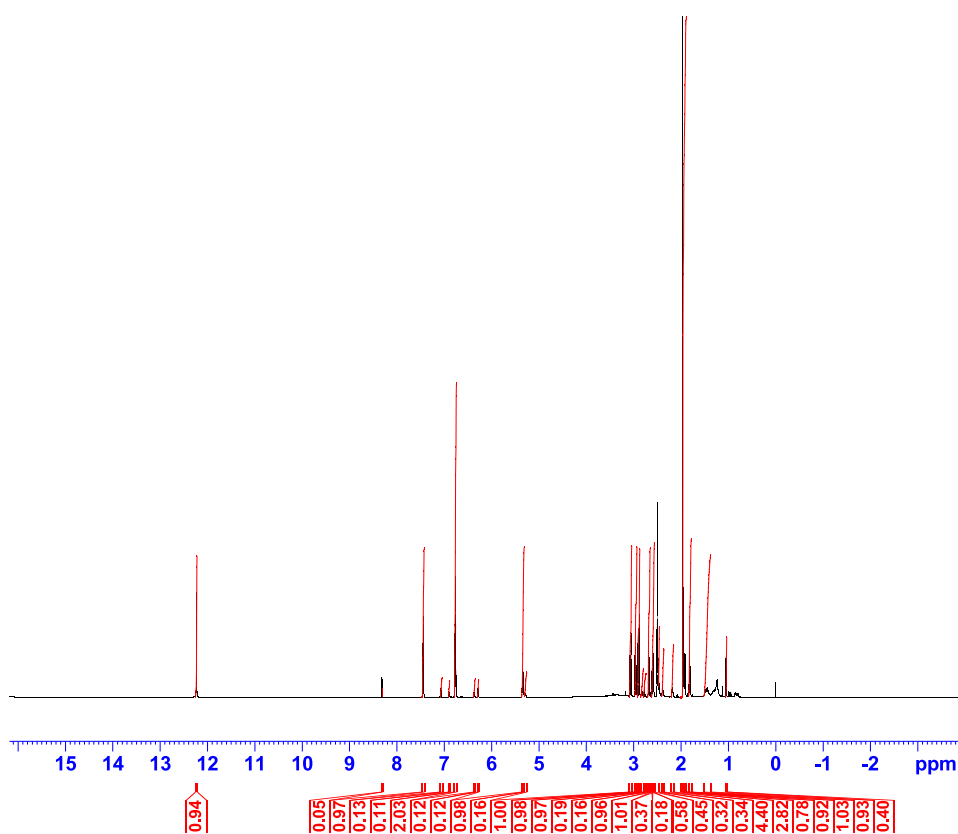

Supplement: S3 Fig — (PDF) [file pone.0308684.s004.pdf]

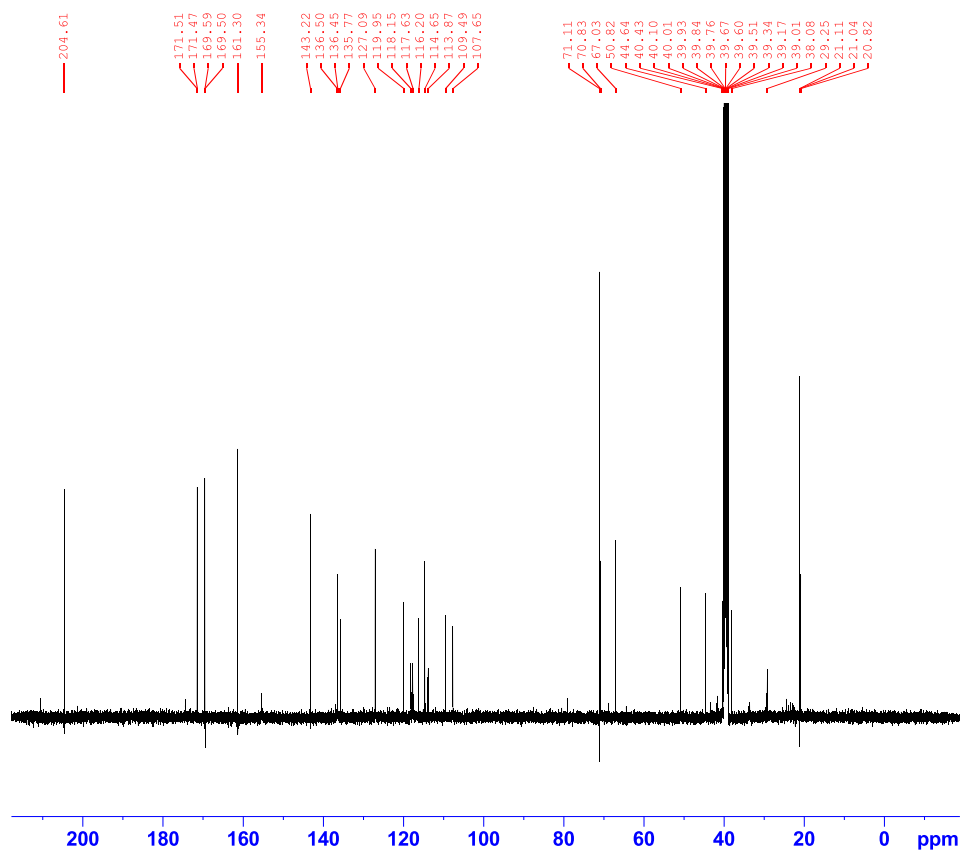

Supplement: S4 Fig — (PDF) [file pone.0308684.s005.pdf]

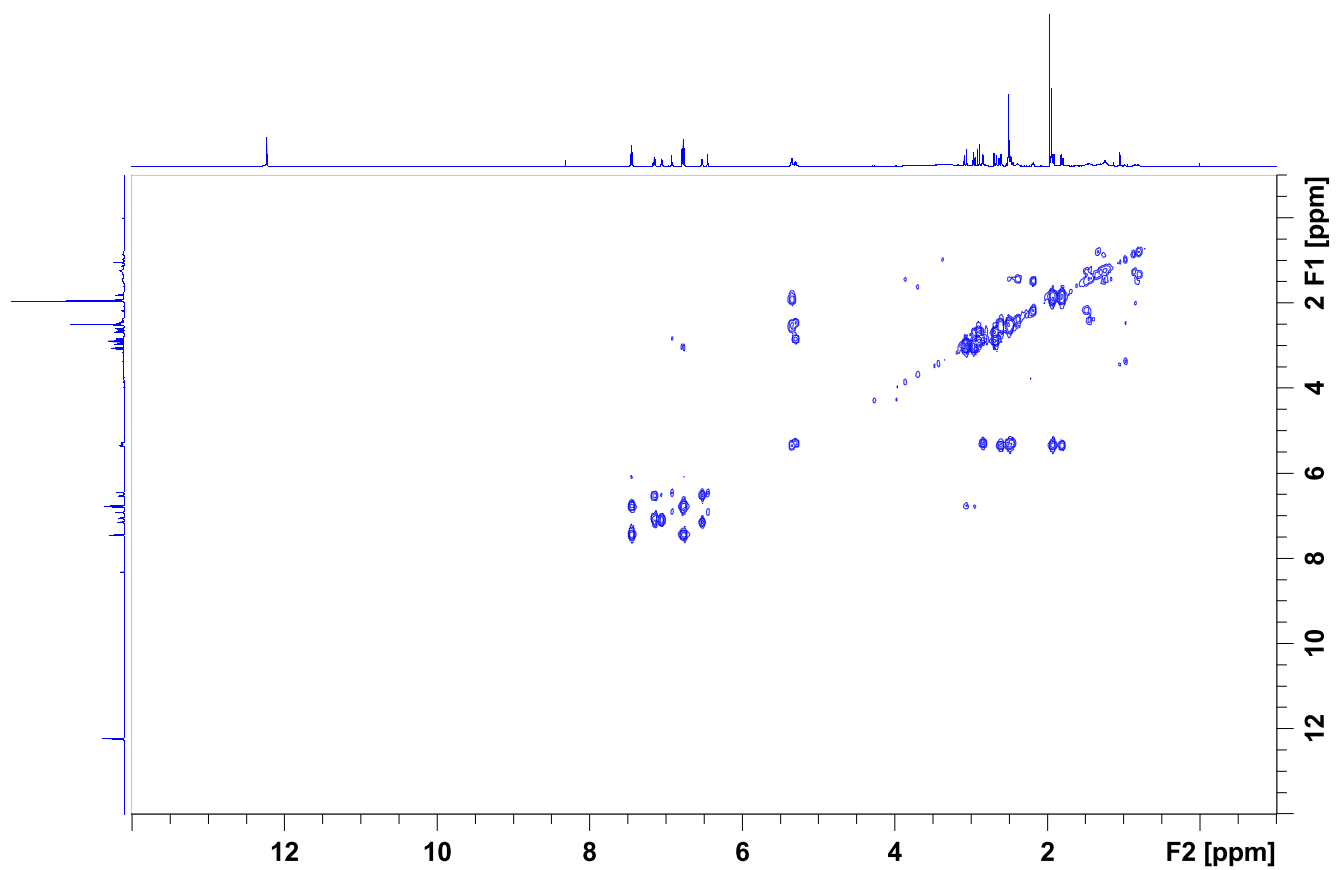

Supplement: S5 Fig — (PDF) [file pone.0308684.s006.pdf]

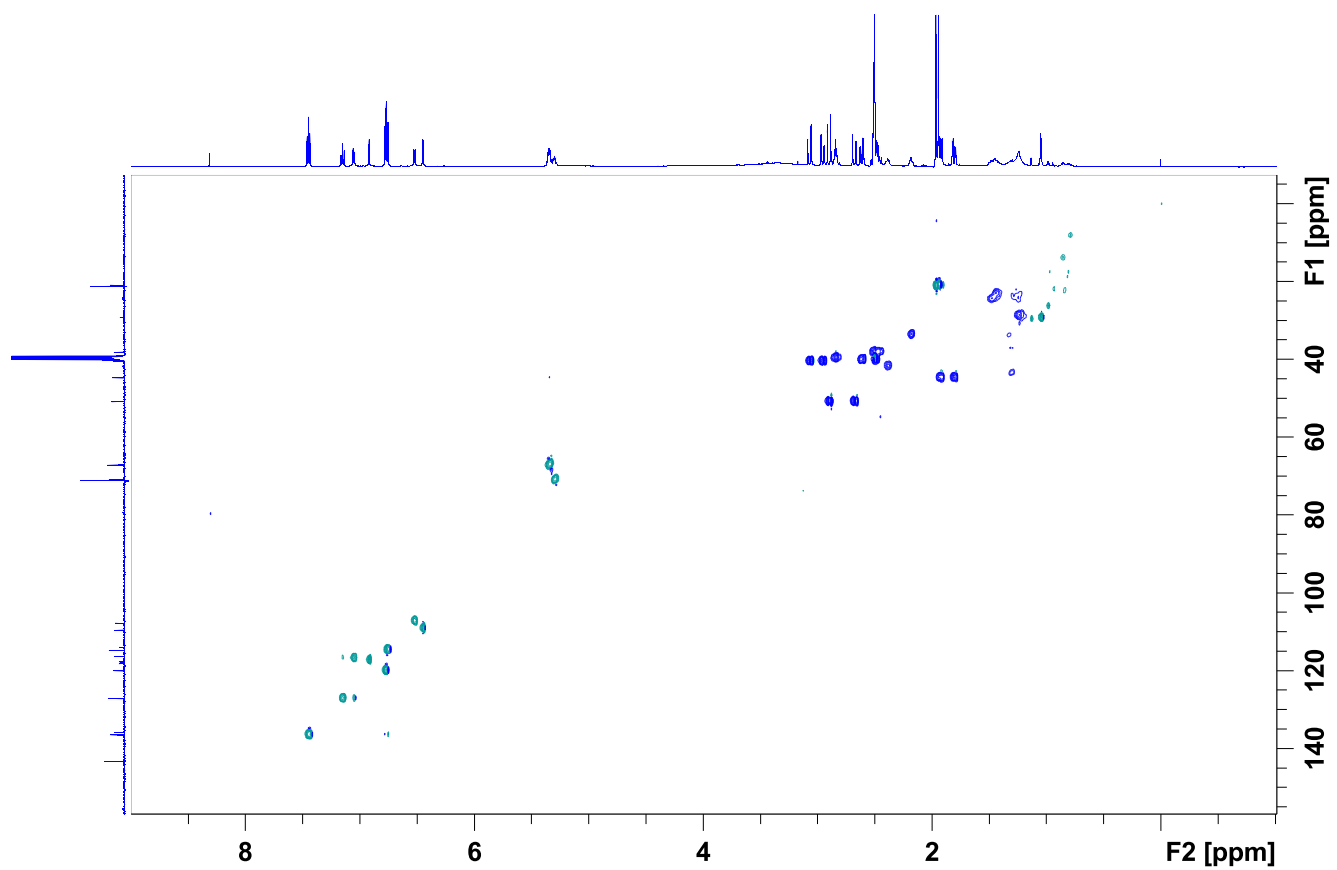

Supplement: S6 Fig — (PDF) [file pone.0308684.s007.pdf]

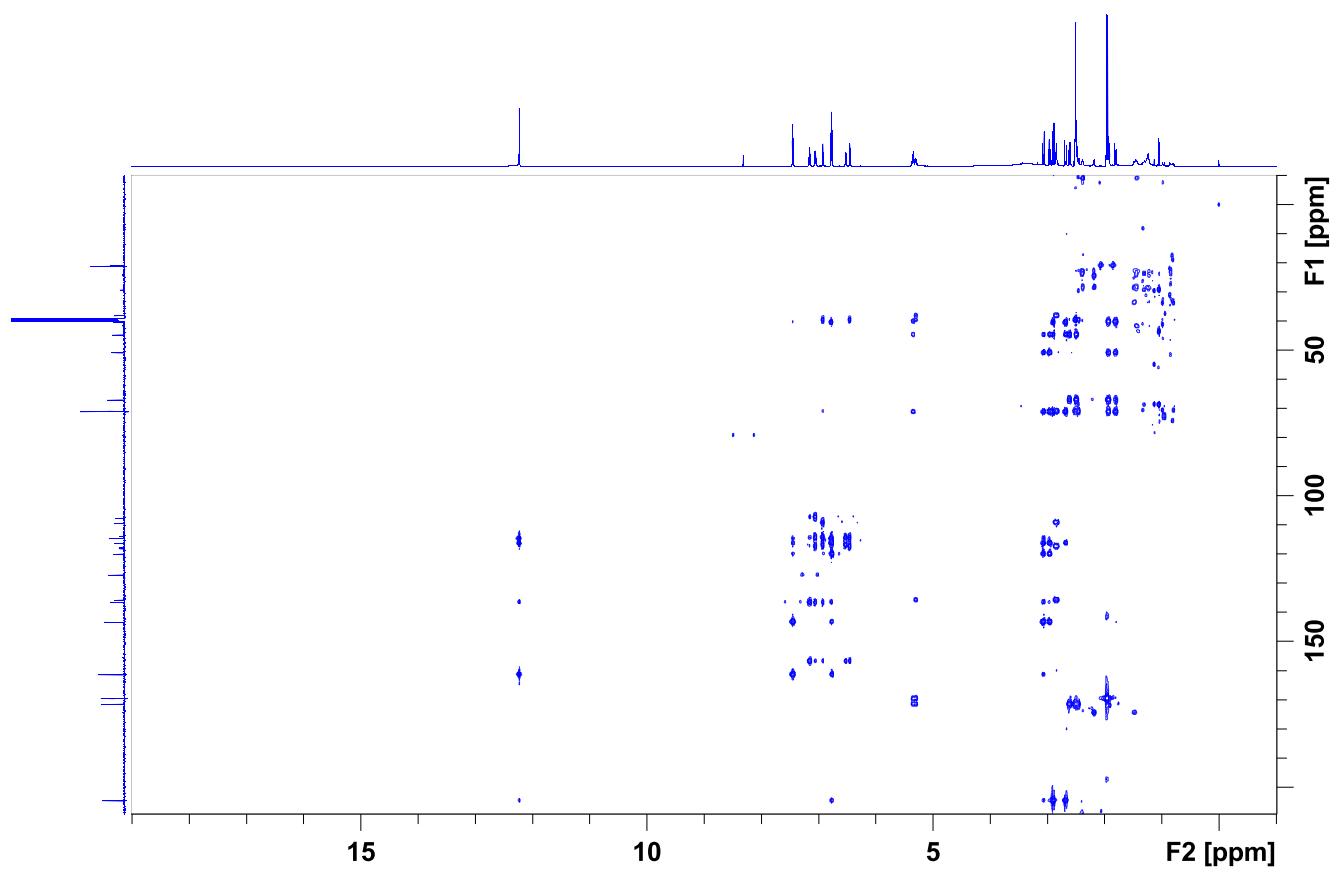

Supplement: S7 Fig — (PDF) [file pone.0308684.s008.pdf]

A

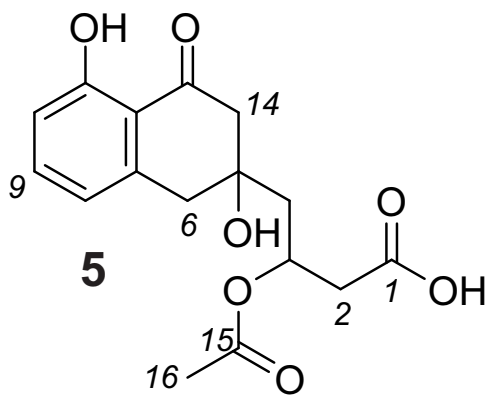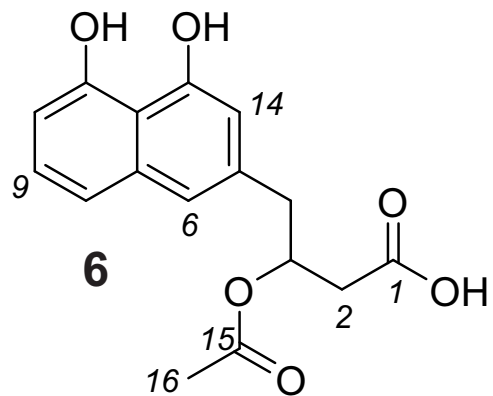

B

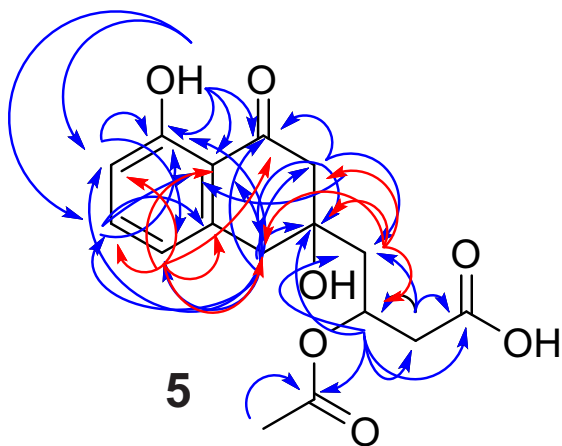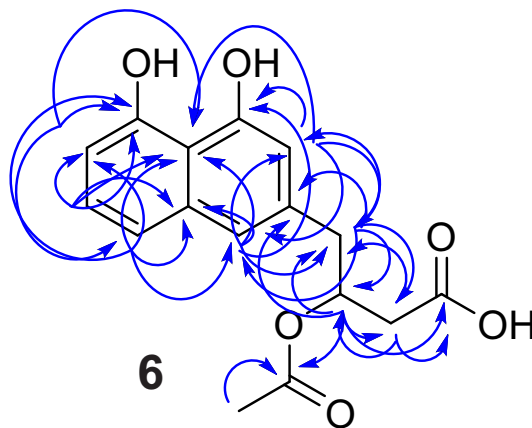

C

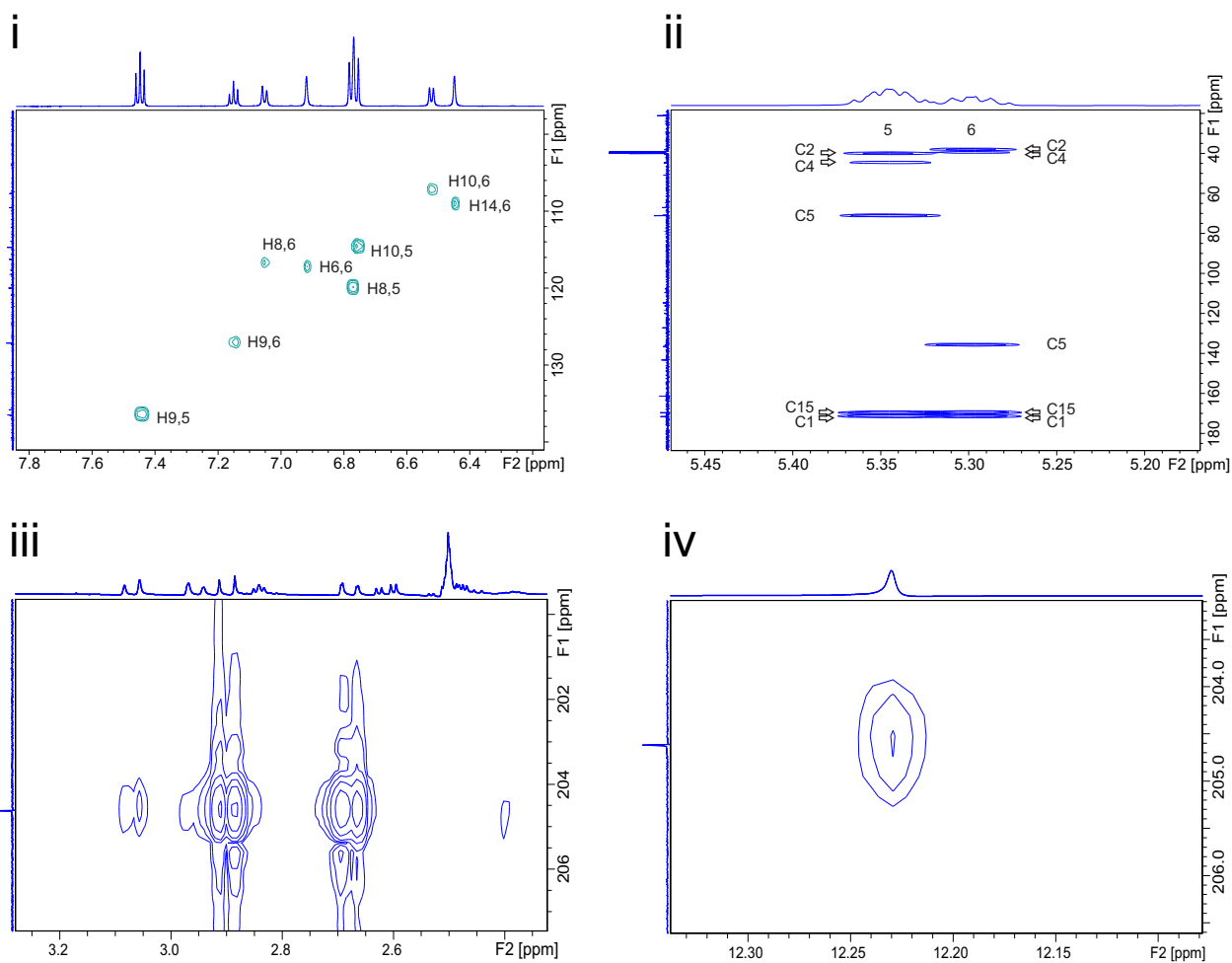

v

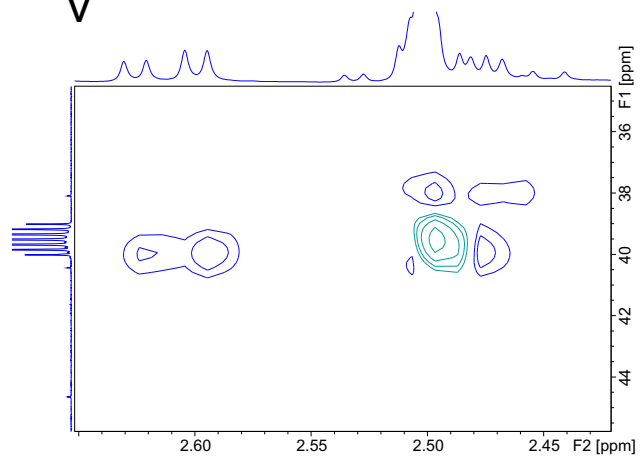

vi

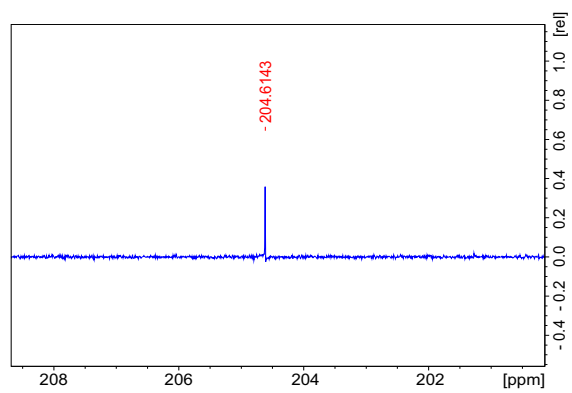

Supplement: S8 Fig — (A) Molecular structures and numberings of 5 and 6; (B) HMBC correlations in the compounds. Two-colored lines, red and blue, are used to show the many correlations clearly; (C) Relevant features of the compound are shown in the selected spectra. The atoms and the compounds 5 or 6 are labeled. i) HSQC spectrum indicating the aromatic hydrogens in both compounds. Compound 5 has three aromatic hydrogens linked to carbons, all coupled. Compound 6 has five aromatic hydrogens linked to carbons, of which three are visibly coupled (J = 0.7, 7.6, 8.3), and a coupling between the other two was indicated (J = 0.8); ii) HMBC spectrum showing the correlations of H3 (left for 5 and right for 6). These signals show the major differences between these two compounds. Both compounds have correlations to C1, C2, C4, C5, and C15, but the shifts are different for C5 and C4 (δ 71.1 vs. δ 135.8, δ 44.6 vs. δ 39.5, respectively). The C5 shift in 5 indicates an attached OH-group, whereas the shift δ 135.8 for 6 corresponds to an aromatic quaternary carbon. The change of the C4 shift is due to the structural difference at C5; iii) and iv) HMBC spectra showing the correlations of H6 and C13 and OH11 and C13 in 5, respectively; v) HSQC spectrum showing the correlations of H2 to C2 in 5 (δ 2.62 and 2.48 to 40.0) and 6 (δ 2.50 and 2.47 to 38.1), which are under the solvent peaks; vi) The carbon peak of C13 (δ 204.6) in 5. In that region, only the peak for 5 is visible. No corresponding peak is observed for 6. (PDF) [file pone.0308684.s009.pdf]

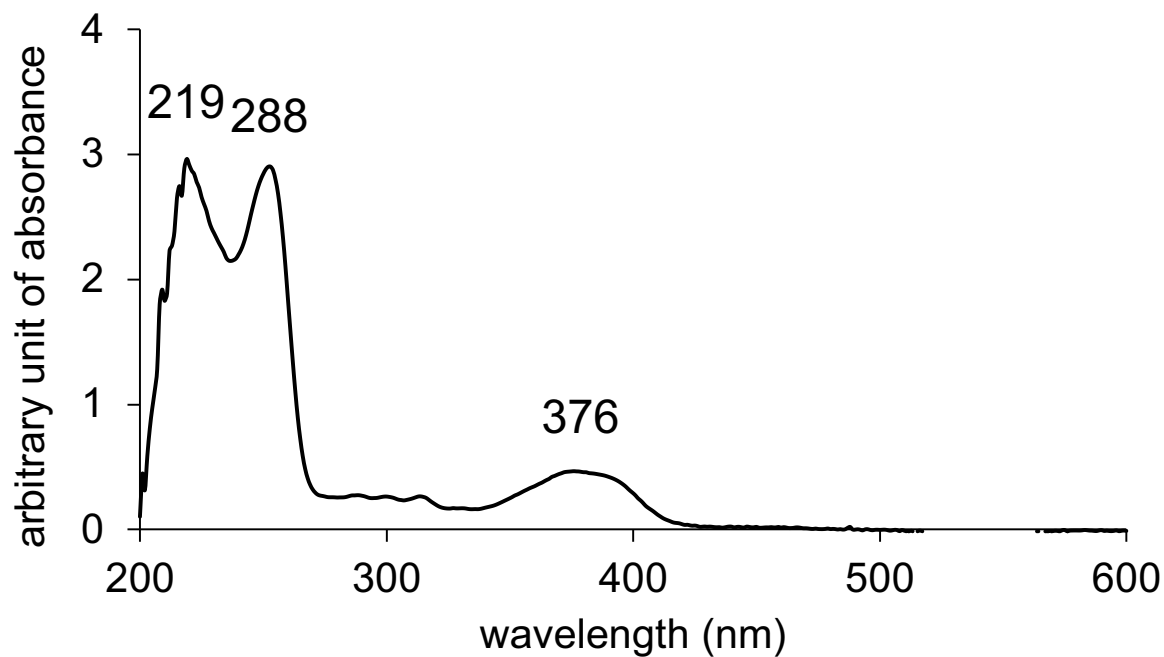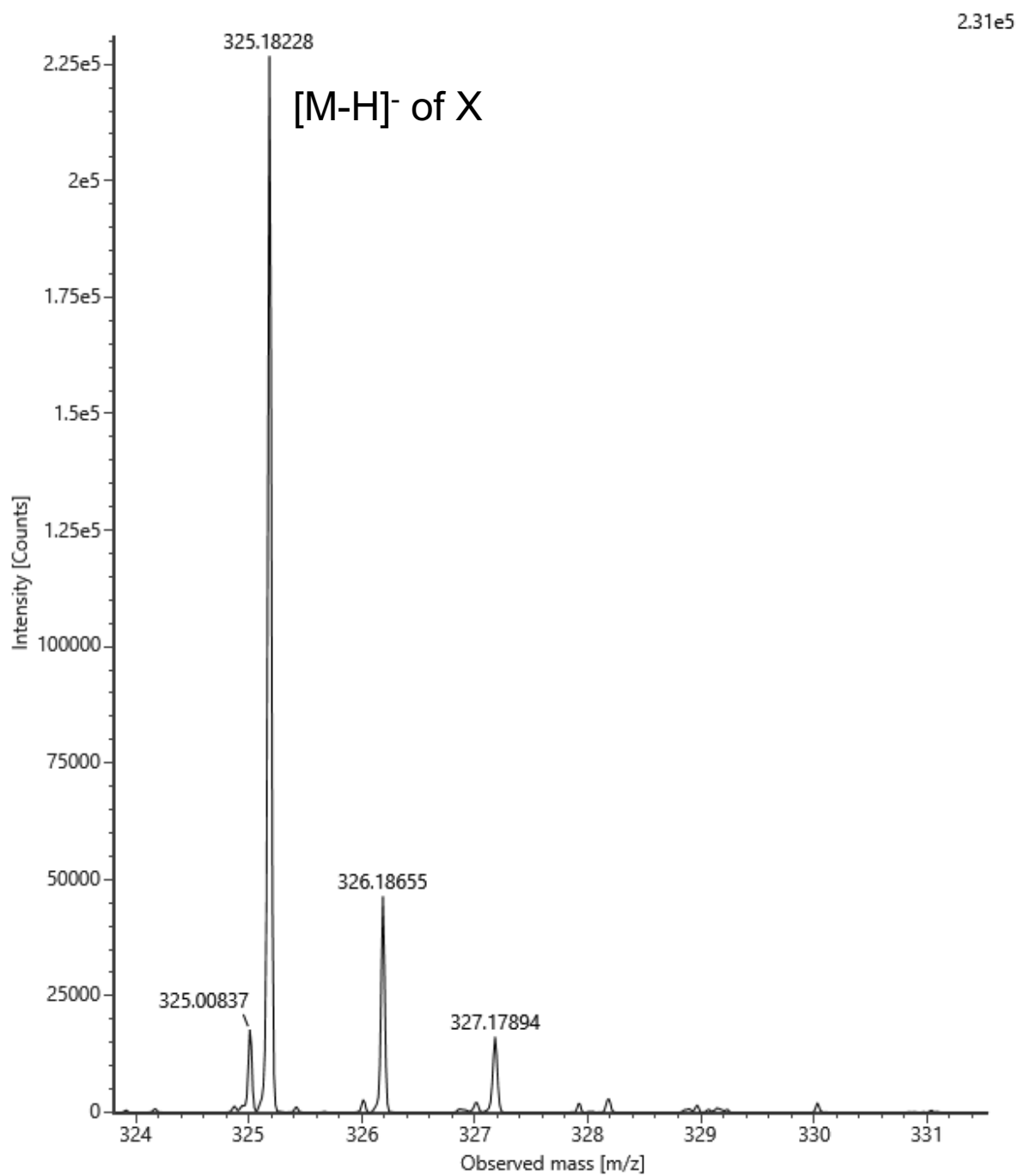

Supplement: S9 Fig — UV-vis spectrum (top) and HRMS (ESI) in negative ionization mode (bottom) of compound X. The featured absorbance wavelengths (nm) are labeled. Observed HRMS ([M-H]-, ESI) m/z: 325.18228. Predicted molecular formula: C11H22N10O2 and C10H26N6O6. (PDF) [file pone.0308684.s010.pdf]
